# Supplementary material for: Spatio-molecular gene expression reflects dorsal anterior cingulate cortex structure and function in the human brain
Source: bioRxiv. 2025 Jul 17:2025.07.14.664821. Preprint. [Version 1] doi: 10.1101/2025.07.14.664821 (PMC12338615; doi:10.1101/2025.07.14.664821)
Supplement: Supplement 1 [file NIHPP2025.07.14.664821v1-supplement-1.pdf]

bioRxiv preprint doi: https://doi.org/10.1101/2025.07.14.664821; this version posted July 17, 2025. The copyright holder for this preprint (which was not certified by peer review) is the author/funder, who has granted bioRxiv a license to display the preprint in perpetuity. It is made available under aCC-BY 4.0 International license.

Supplementary Figures

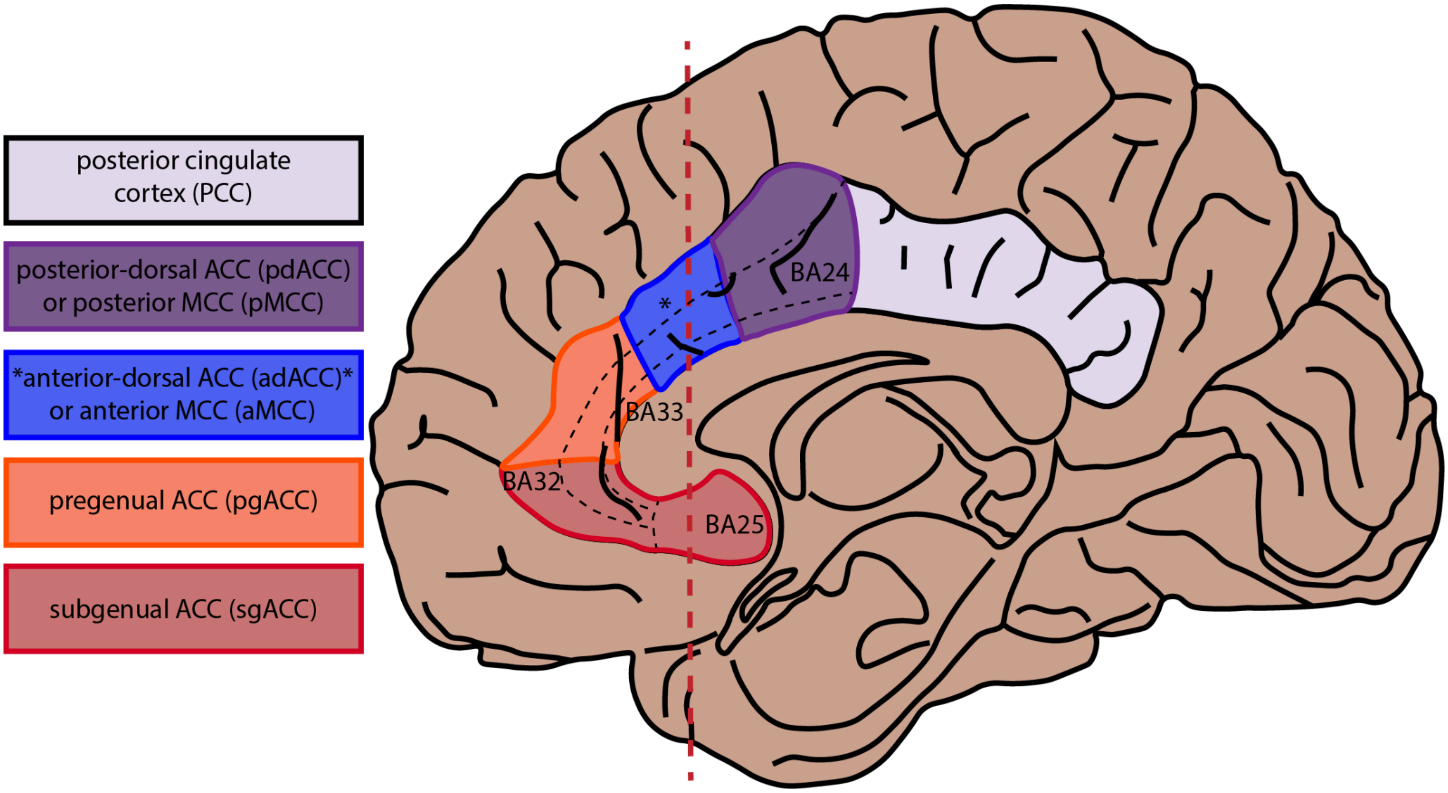

Supplementary Fig. 1. **Region annotations across the ACC.**

Illustration of the midsagittal view of the human brain indicating five subdivisions of the cingulate cortex (CC): red - subgenual anterior CC (sgACC); orange - pregenual anterior CC (pgACC); blue - anterior-dorsal anterior CC (adACC) or anterior mid CC (aMCC); purple - posterior-dorsal anterior CC (pdACC) or posterior mid CC (pMCC); lavender - posterior CC (PCC). Approximate boundaries of Brodmann areas (BAs) 25, 33, 24, and 32 are indicated by black dashed lines across the CC. Red vertical dashed line indicates the level at which the coronal slabs were selected for this study, targeting the adACC/aMCC as the region of interest (blue*).

bioRxiv preprint doi: https://doi.org/10.1101/2025.07.14.664821; this version posted July 17, 2025. The copyright holder for this preprint (which was not certified by peer review) is the author/funder, who has granted bioRxiv a license to display the preprint in perpetuity. It is made available under aCC-BY 4.0 International license.

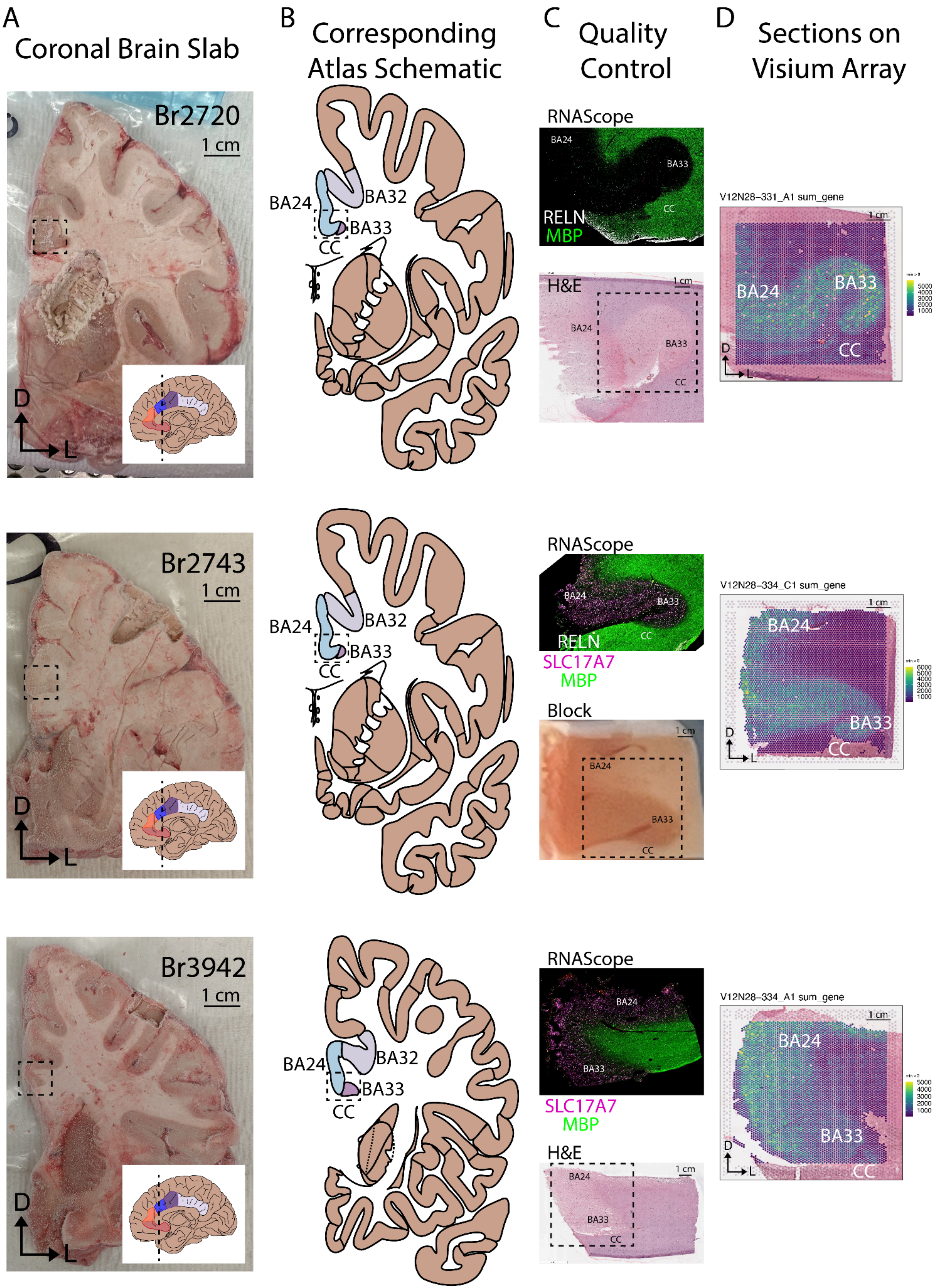

bioRxiv preprint doi: https://doi.org/10.1101/2025.07.14.664821; this version posted July 17, 2025. The copyright holder for this preprint (which was not certified by peer review) is the author/funder, who has granted bioRxiv a license to display the preprint in perpetuity. It is made available under aCC-BY 4.0 International license.

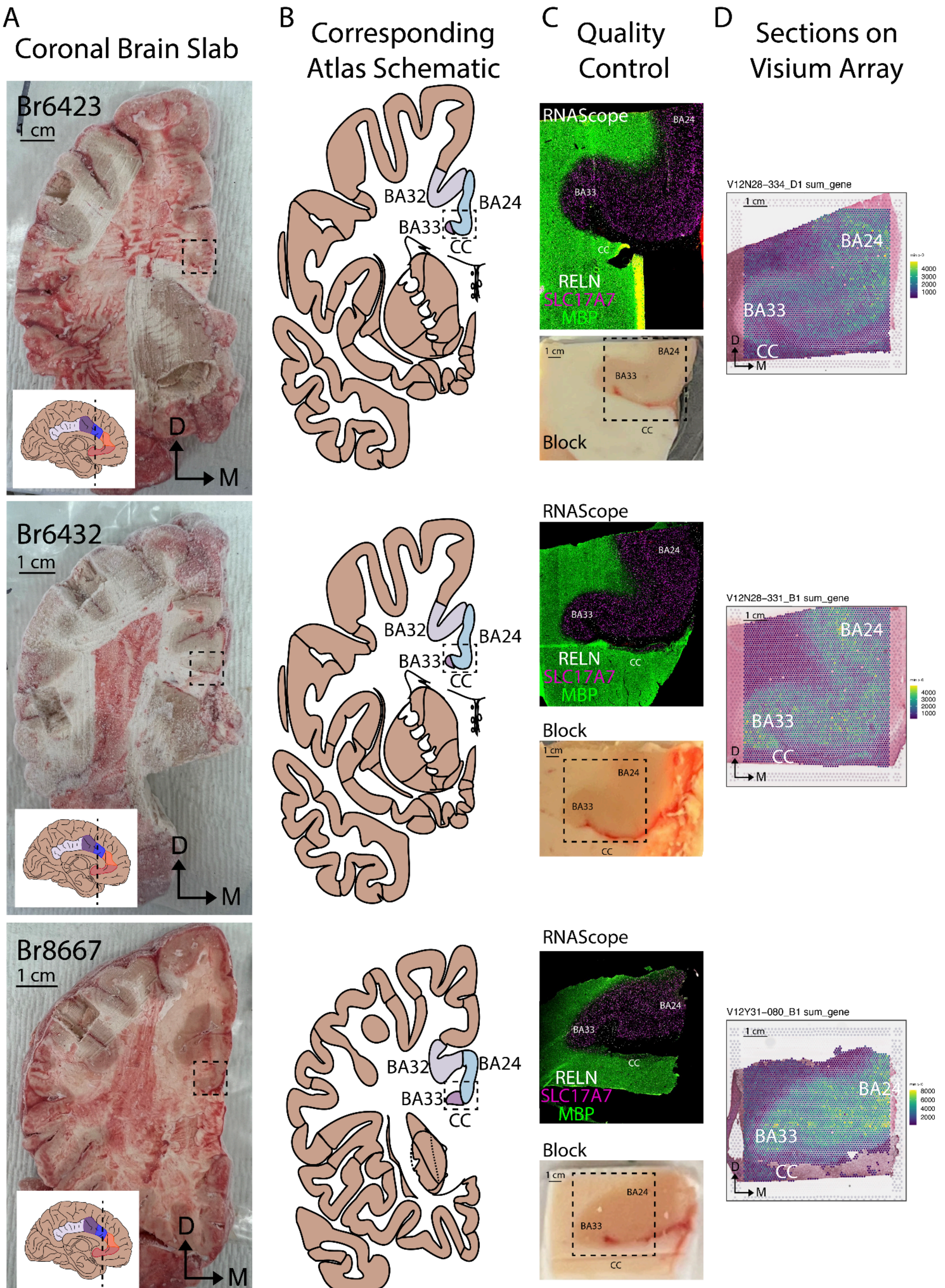

bioRxiv preprint doi: https://doi.org/10.1101/2025.07.14.664821; this version posted July 17, 2025. The copyright holder for this preprint (which was not certified by peer review) is the author/funder, who has granted bioRxiv a license to display the preprint in perpetuity. It is made available under aCC-BY 4.0 International license.

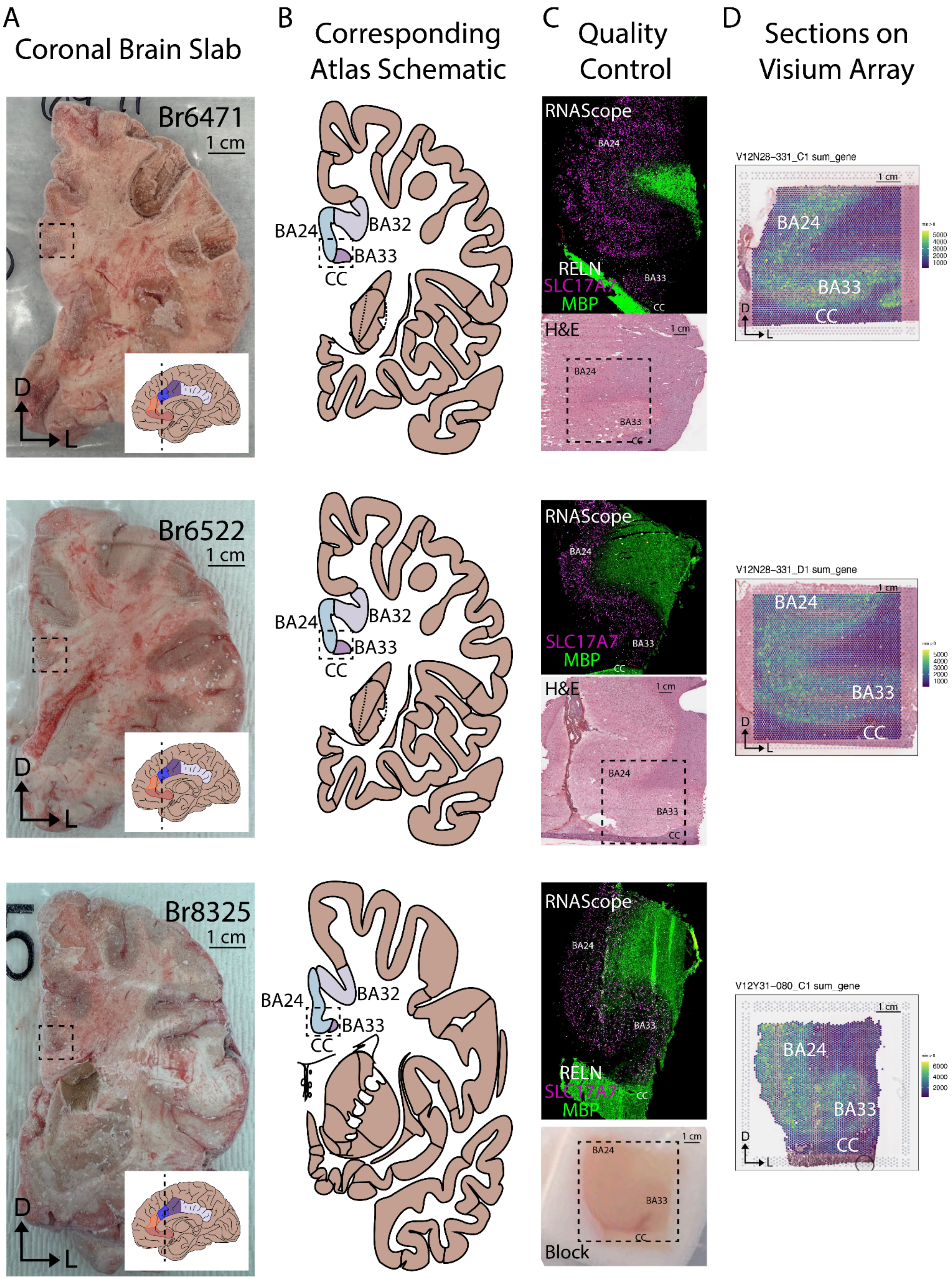

bioRxiv preprint doi: https://doi.org/10.1101/2025.07.14.664821; this version posted July 17, 2025. The copyright holder for this preprint (which was not certified by peer review) is the author/funder, who has granted bioRxiv a license to display the preprint in perpetuity. It is made available under aCC-BY 4.0 International license.

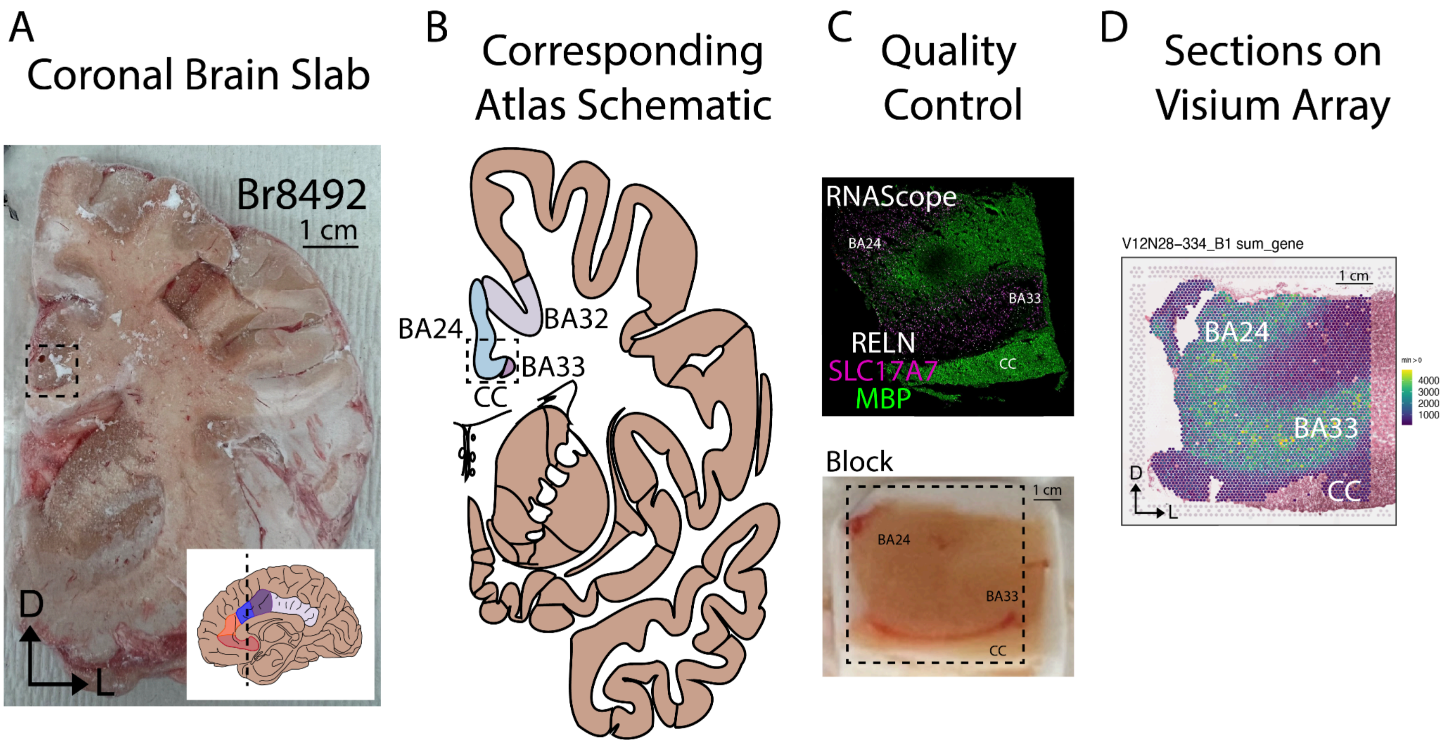

Supplementary Fig. 2. **Neuroanatomical validation of dACC location across 10 donors.**
(**A**) Fresh-frozen coronal brain slabs from the n=10 neurotypical control donors. Dashed boxes indicate the location of the dissected blocks in (**C**). Inset depicts a midsagittal view of the brain with five subdivisions of the cingulate cortex (red - subgenual region; orange - pregenual region; blue - anterior-dorsal ACC or anterior MCC; purple - posterior-dorsal ACC or posterior MCC; lavender - posterior cingulate cortex). Dashed line across the midsagittal schematic indicates the level of the coronal slab. Neuroanatomical orientation is indicated by arrows: D - dorsal; L - lateral; M - medial.

(**B**) Brain atlas schematic corresponding to the level of the coronal cut of the brain slab in (**A**). Brodmann areas (BAs), BA33, BA24, and BA32, which make up the cingulate cortex, are indicated. CC - corpus callosum. Dashed boxes indicate the location of the dissected blocks in (**C**).

(**C**) Quality control experiment summary for each dissected brain block. RNAScope experiments with probes marking *MBP* (white matter), *SLC17A7* (excitatory neurons), and/or *RELN* (Layer 1), are presented in green, pink, and white, respectively. Images of H&E or unprocessed brain blocks with all relevant structures of the block are indicated: BA24, BA33, CC - corpus callosum. Dashed boxes indicate the area of tissue placed on the Visium array in (**D**).

(**D**). Sections from each donor, as placed on Visium arrays, rotated to match the orientation of each slab. BAs are indicated; CC - corpus callosum. Neuroanatomical orientation is indicated by arrows: D - dorsal; L - lateral; M - medial.

bioRxiv preprint doi: https://doi.org/10.1101/2025.07.14.664821; this version posted July 17, 2025. The copyright holder for this preprint (which was not certified by peer review) is the author/funder, who has granted bioRxiv a license to display the preprint in perpetuity. It is made available under aCC-BY 4.0 International license.

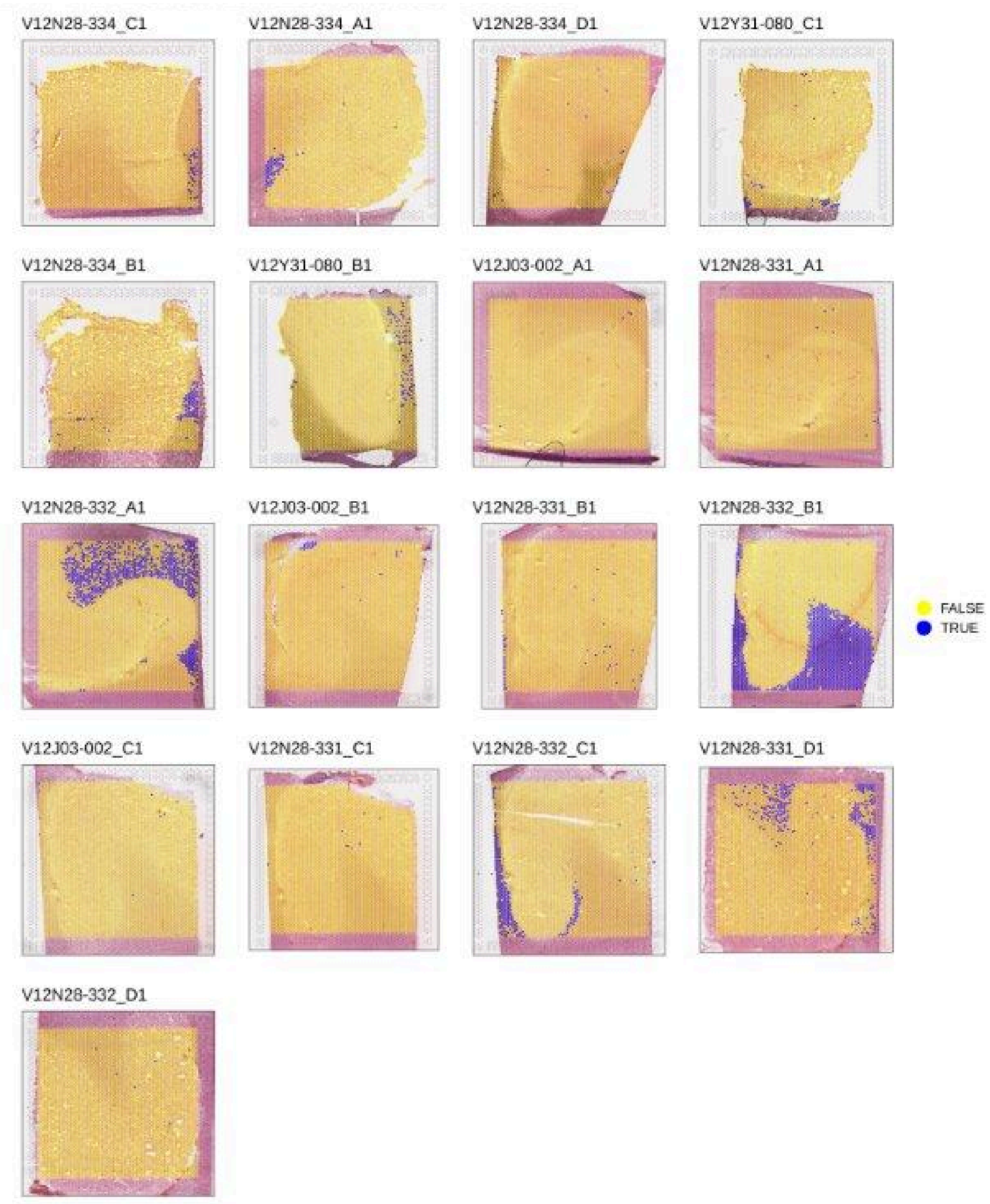

Supplementary Fig. 3. Spot plots per sample showing 3x median-absolute-deviation (MAD) threshold for spots with low library size and/or low number of detected genes. The spot plot is overlaid on the histology image. Blue color represents spots that are 3 MADs below the median library size and/or median number of detected genes for each sample. If the 3 MADs criteria was used to remove spots, then these spots would have been removed. As described in **Methods**, the criteria from **Supplementary Fig. 4** was used to remove spots instead.

bioRxiv preprint doi: https://doi.org/10.1101/2025.07.14.664821; this version posted July 17, 2025. The copyright holder for this preprint (which was not certified by peer review) is the author/funder, who has granted bioRxiv a license to display the preprint in perpetuity. It is made available under aCC-BY 4.0 International license.

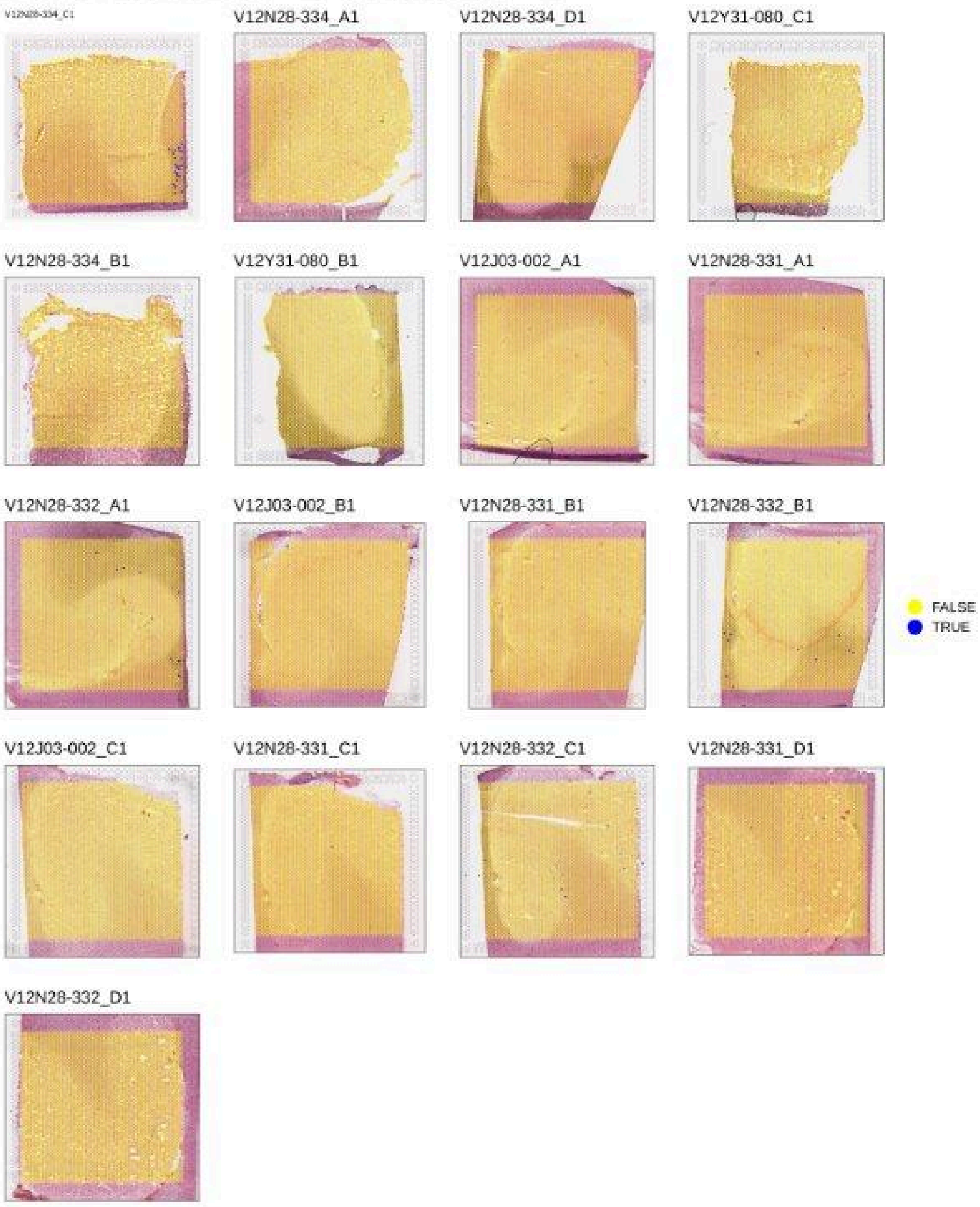

Supplementary Fig. 4. Spot plots per sample showing extreme filter for spots with library size less than 20 and/or number of detected genes less than 20. The spot plot is overlaid on the histology image. Blue color represents spots that have library size less than 20 and/or number of detected genes less than 20; these spots were removed before proceeding with downstream analyses.

bioRxiv preprint doi: https://doi.org/10.1101/2025.07.14.664821; this version posted July 17, 2025. The copyright holder for this preprint (which was not certified by peer review) is the author/funder, who has granted bioRxiv a license to display the preprint in perpetuity. It is made available under aCC-BY 4.0 International license.

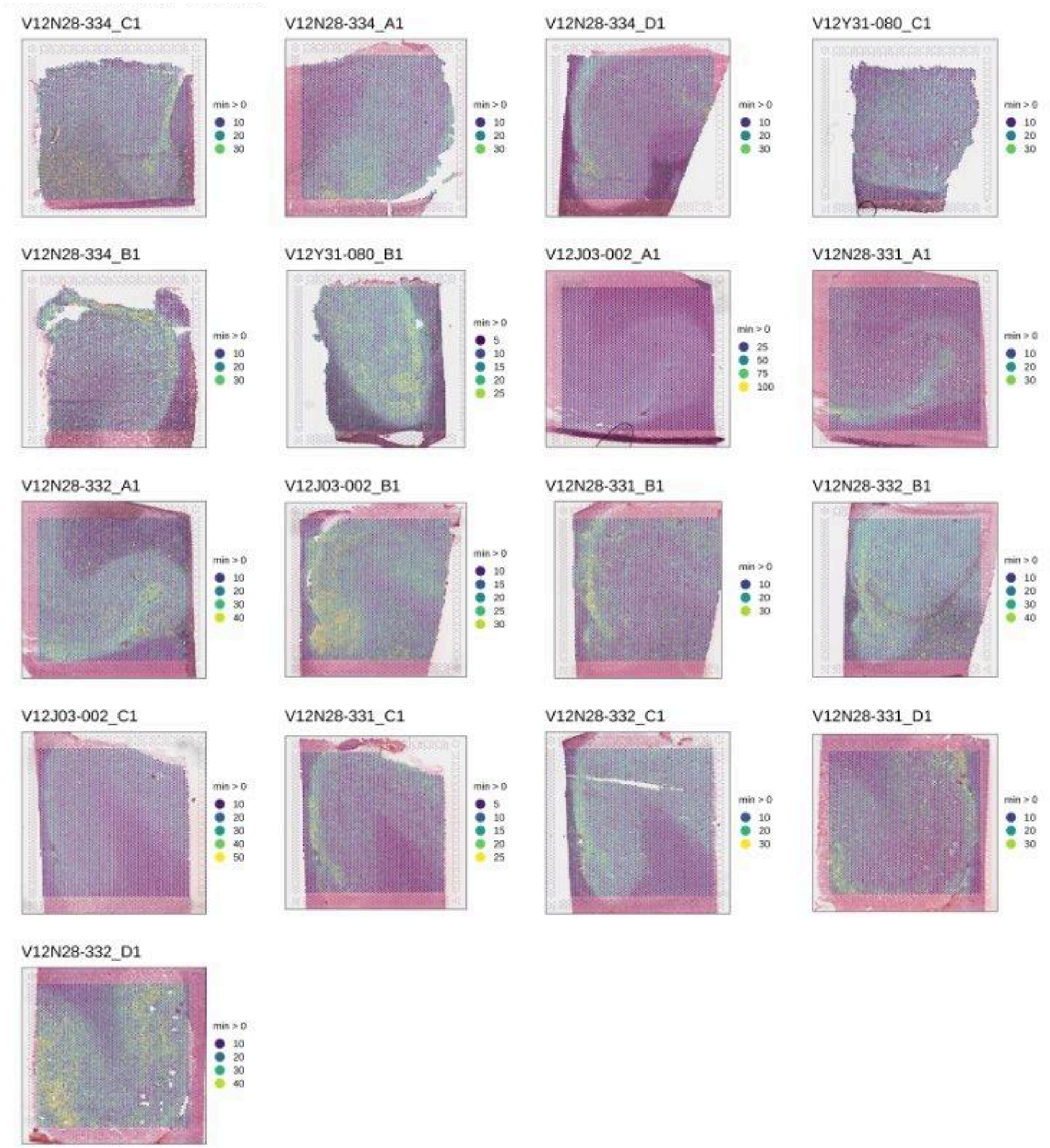

Supplementary Fig. 5. Spot plots per sample showing mitochondrial percentage for each spot. The spot plot is overlaid on the histology image. Color represents the mitochondrial percentage for each spot.

bioRxiv preprint doi: https://doi.org/10.1101/2025.07.14.664821; this version posted July 17, 2025. The copyright holder for this preprint (which was not certified by peer review) is the author/funder, who has granted bioRxiv a license to display the preprint in perpetuity. It is made available under aCC-BY 4.0 International license.

nnSVG-Guided PRECAST Clusters k=5-20 (V12N28-334_C1)

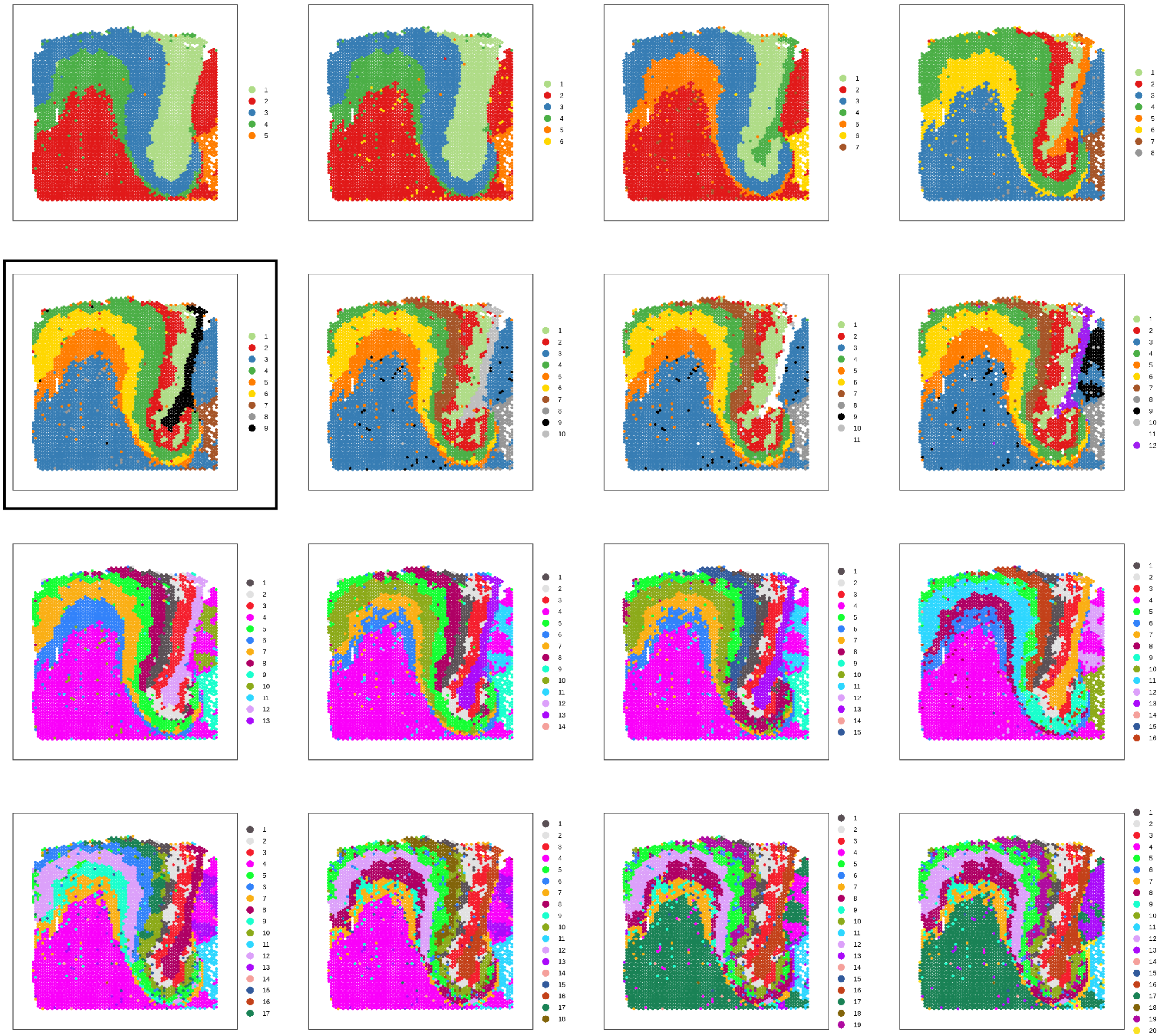

Supplementary Fig. 6. **Spot plots of data-driven `PRECAST` clusters.** Shown on one sample of the dACC SRT data (V12N28-334_C1), the number of clusters increases from 5 to 20 clusters with the descending rows. The `PRECAST` algorithm, guided by `nnSVG` spatially variable genes, was fit with the given number of clusters. Color represents the cluster label of each spot for the given `PRECAST` clustering algorithm. Note that the colors do not necessarily correspond to the same domain across clustering resolutions. The black box highlights the final clusters (*k*=9) selected for downstream analyses.

bioRxiv preprint doi: https://doi.org/10.1101/2025.07.14.664821; this version posted July 17, 2025. The copyright holder for this preprint (which was not certified by peer review) is the author/funder, who has granted bioRxiv a license to display the preprint in perpetuity. It is made available under aCC-BY 4.0 International license.

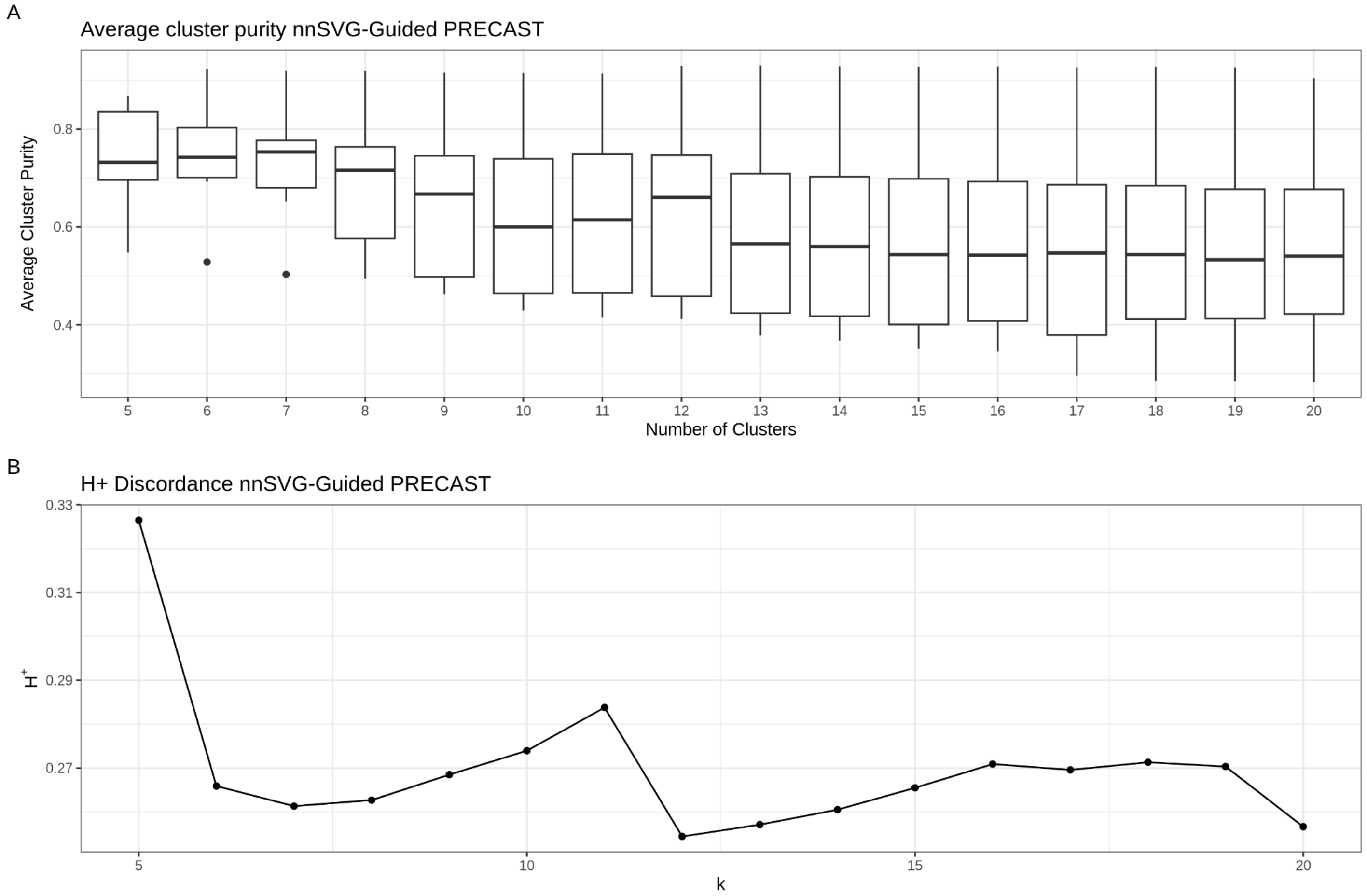

Supplementary Fig. 7. Metrics to evaluate `nnSVG`-guided `PRECAST` clusters of the dACC SRT data. (**A**) Boxplots of average cluster purity (*y*-axis) for each clustering algorithm from `PRECAST` with number of clusters ranging from 5 to 20 (*x*-axis). The purity of each cluster per gene is the proportion of observations in its neighborhood from a different cluster, and the average purity is the average of the purity scores across genes in a specific cluster. Higher cluster purity is desirable. (**B**) Line plot of the H_+ discordance metric (*y*-axis) (computed with `fasthplus`) for each clustering algorithm from `PRECAST` with the number of clusters ranging from *k*=5 to 20 (*x*-axis). This metric measures the discordance of unsupervised clusters, and lower values are desirable.

bioRxiv preprint doi: https://doi.org/10.1101/2025.07.14.664821; this version posted July 17, 2025. The copyright holder for this preprint (which was not certified by peer review) is the author/funder, who has granted bioRxiv a license to display the preprint in perpetuity. It is made available under aCC-BY 4.0 International license.

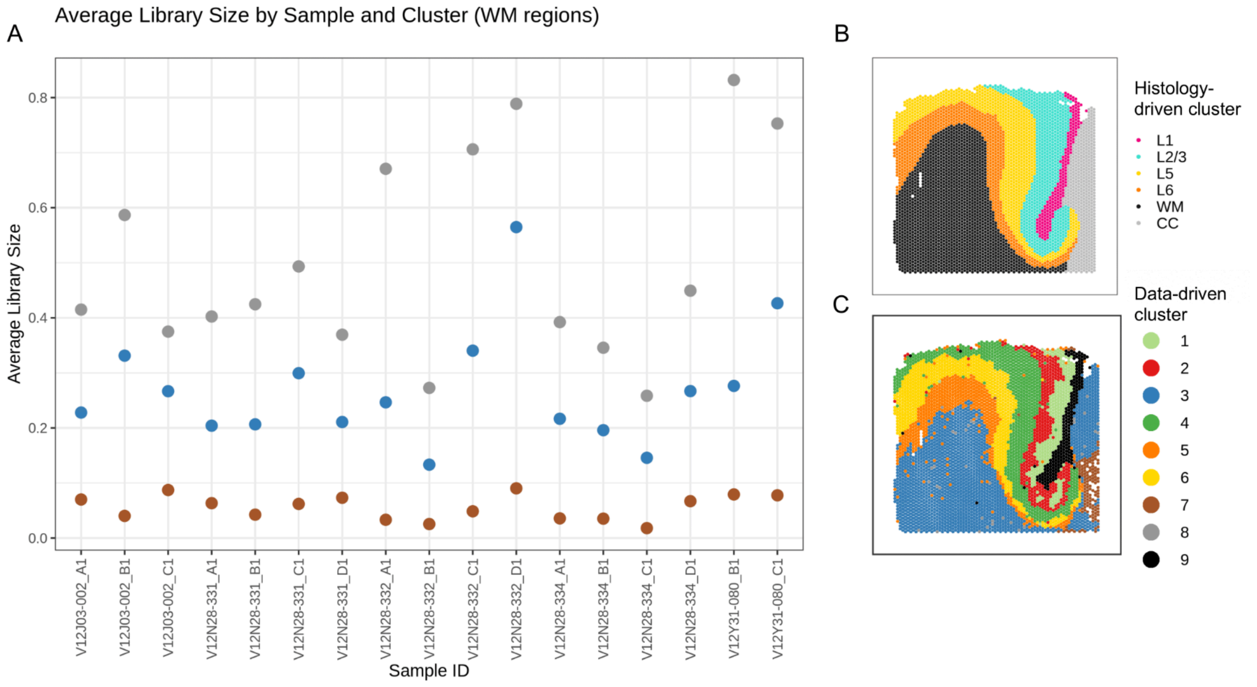

Supplementary Fig. 8. Average library size of white matter `nnSVG`-guided `PRECAST` clusters of the dACC SRT data. Dot plot of average library size (*y*-axis) per sample (*x*-axis) within `PRECAST` clustering algorithm *k*=9. Color represents cluster number, matching with *k*=9 in **Supplementary Fig. 6** (**C**), with an example from sample V12N28-334_C1. (**B**) Domains from sample V12N28-334_C1. Color represents the histology-driven manually annotated spatial domain of each spot.

bioRxiv preprint doi: https://doi.org/10.1101/2025.07.14.664821; this version posted July 17, 2025. The copyright holder for this preprint (which was not certified by peer review) is the author/funder, who has granted bioRxiv a license to display the preprint in perpetuity. It is made available under aCC-BY 4.0 International license.

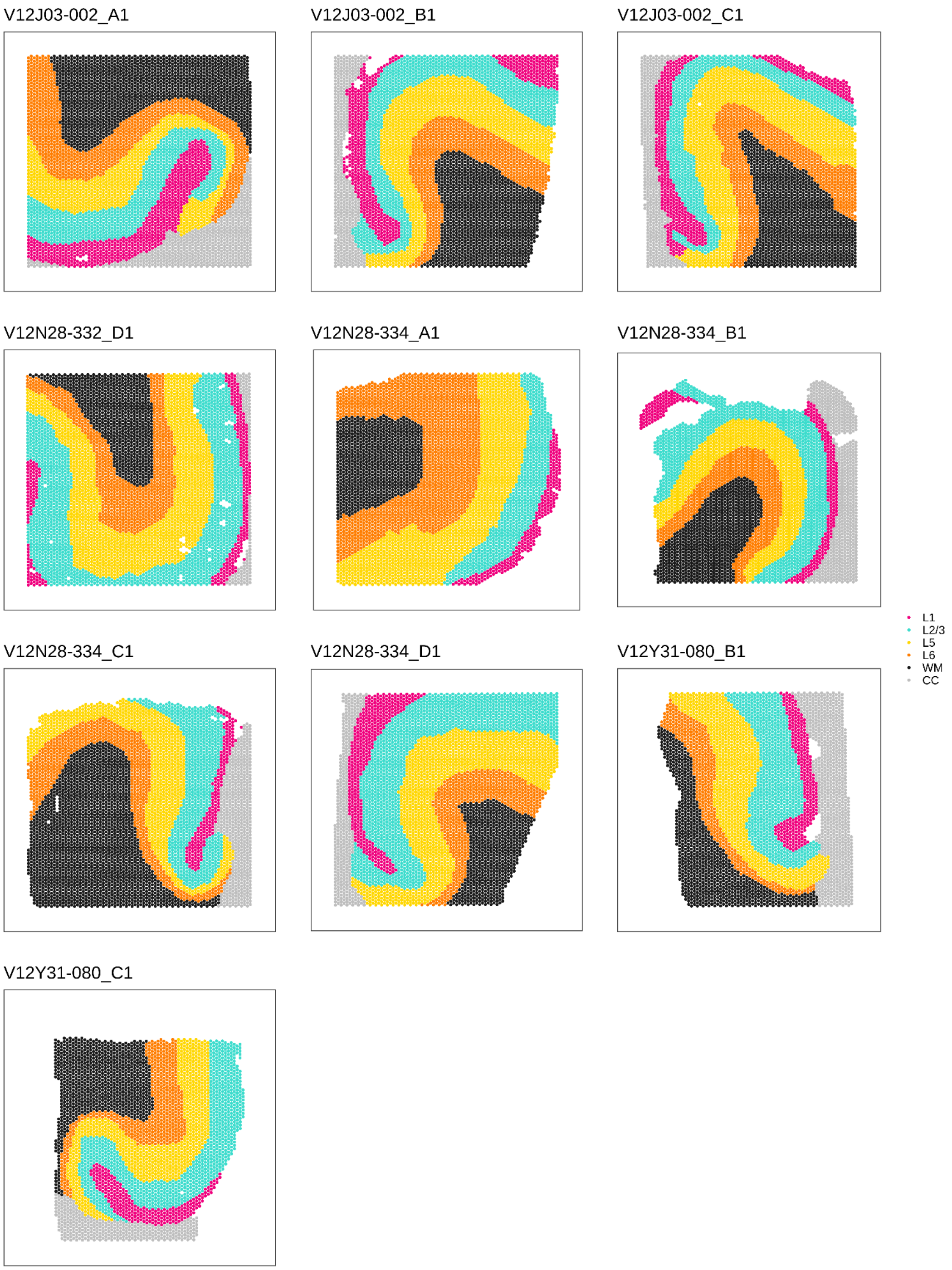

Supplementary Fig. 9. Spot plots showing histology-driven manual annotations in dACC SRT data. For ten samples of the dACC SRT data, one sample for each donor was manually annotated with `Samui Browser` (Sriworarat et al. 2023) based on anatomical features and laminar marker genes. Color represents the histology-driven manually annotated spatial domain of each spot.

bioRxiv preprint doi: https://doi.org/10.1101/2025.07.14.664821; this version posted July 17, 2025. The copyright holder for this preprint (which was not certified by peer review) is the author/funder, who has granted bioRxiv a license to display the preprint in perpetuity. It is made available under aCC-BY 4.0 International license.

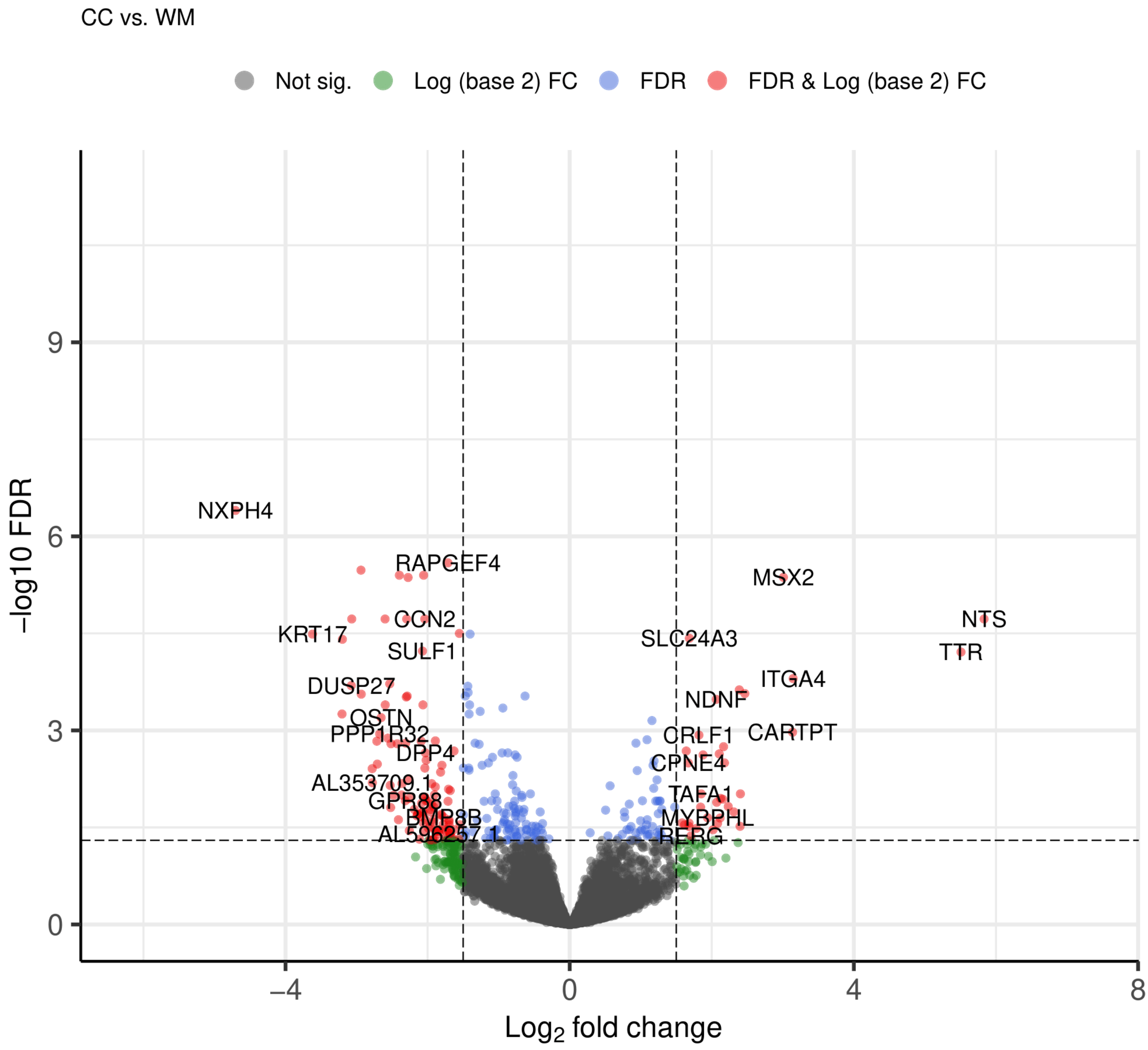

Supplementary Fig. 10. Differential expression of white matter domains from histology-driven manual annotations in dACC SRT data. `EnhancedVolcano` plot shows the DE results for the pairwise model pseudobulked test for the CC (corpus callosum) dACC SRT manual annotation compared to the WM (white matter) dACC SRT manual annotation. Each point is a gene with its log fold-change (logFC) (*x*-axis) and statistical significance (*y*-axis). Statistical significance is measured with negative log-transformation of FDR-adjusted *p*-values. Color indicates categorization of each gene; red represents statistically significant with FDR < 0.05 and absolute value of logFC > 1, blue represents statistically significant with FDR < 0.05 only, grey represents not statistically significant with FDR >= 0.05, and green represents absolute value of logFC > 1 only.

bioRxiv preprint doi: https://doi.org/10.1101/2025.07.14.664821; this version posted July 17, 2025. The copyright holder for this preprint (which was not certified by peer review) is the author/funder, who has granted bioRxiv a license to display the preprint in perpetuity. It is made available under aCC-BY 4.0 International license.

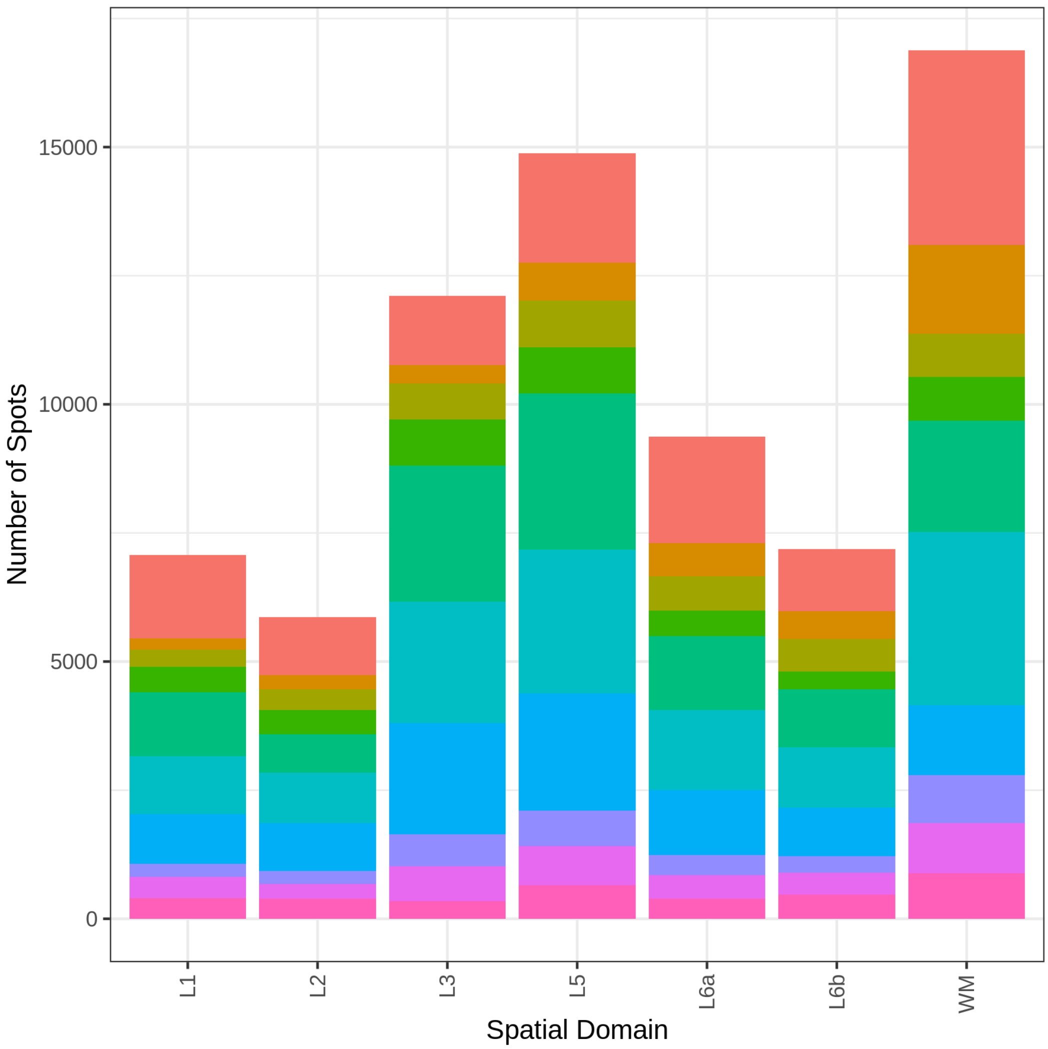

Supplementary Fig. 11. **Brain donor composition of dACC SRT spatial domains.** Barplot displays the number of spots (*y*-axis) in each spatial domain (*x*-axis) in the dACC SRT data. Color represents the proportion of spots in each spatial domain coming from each donor.

bioRxiv preprint doi: https://doi.org/10.1101/2025.07.14.664821; this version posted July 17, 2025. The copyright holder for this preprint (which was not certified by peer review) is the author/funder, who has granted bioRxiv a license to display the preprint in perpetuity. It is made available under aCC-BY 4.0 International license.

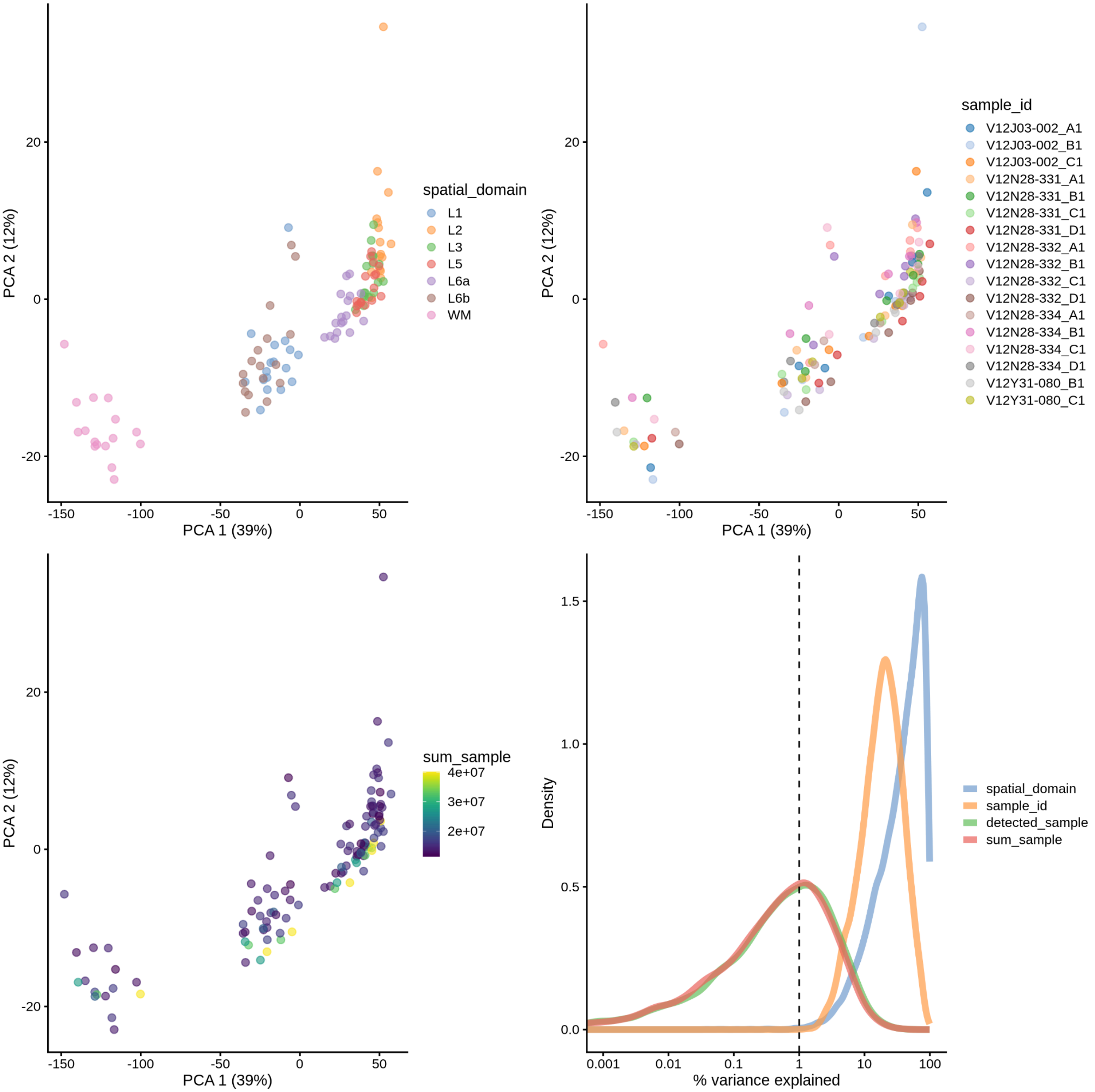

Supplementary Fig. 12. **Principal components analysis (PCA) of pseudobulked dACC SRT data.** The first three plots show the score plots of the first two (PCs) of the pseudobulked dACC SRT spatial domains. Each score plot is colored by spatial domain, sample id, and total UMI counts per sample, respectively. The fourth plot, made with `scater (McCarthy et al. 2017)`, shows the percent variance explained by spatial domain, sample id, total UMI counts per sample, and total detected genes per sample.

bioRxiv preprint doi: https://doi.org/10.1101/2025.07.14.664821; this version posted July 17, 2025. The copyright holder for this preprint (which was not certified by peer review) is the author/funder, who has granted bioRxiv a license to display the preprint in perpetuity. It is made available under aCC-BY 4.0 International license.

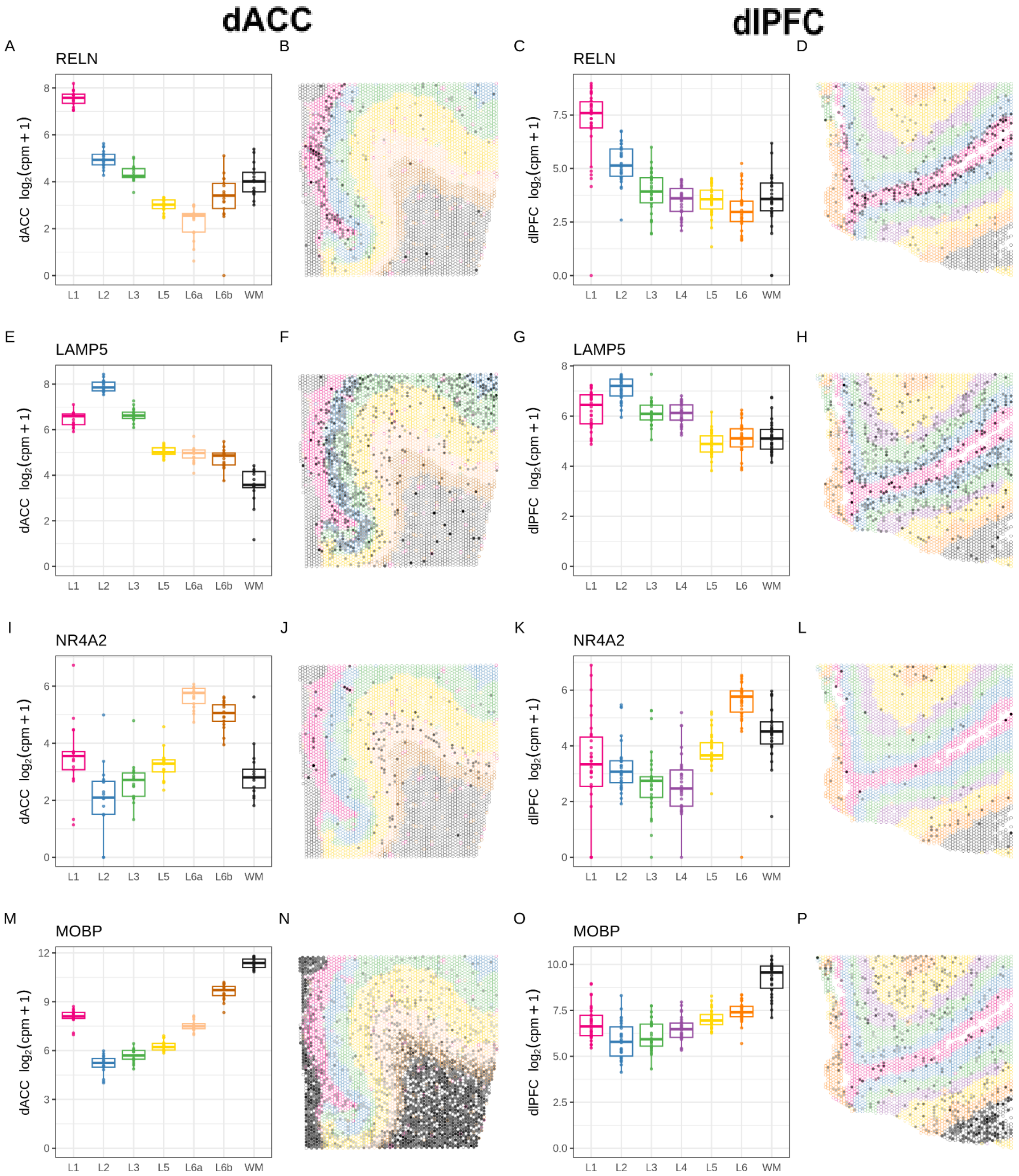

Supplementary Fig. 13. **Spatial domain layer markers in paired dACC and dlPFC SRT data.** Each row shows information for one gene, in order, *RELN, LAMP5, NR4A2, MOBP.* Each column displays a similar style of plot. First column (**A, E, I, M**): The *y*-axis displays $\log_2$(counts per million + 1) expression (computed manually) for each spatial domain (*x*-axis) in the pseudobulked dACC SRT data. Color represents the spatial domain. Second column (**B, F, J, N**): `escheR` spot plot of dACC Visium capture area from donor Br6432 (sample ID: V12N28-331_B1) with spots colored by the dACC spatial domains. Fill represents $\log_2$-normalized expression per spot. Third column (**C, G, K, O**): The *y*-axis displays $\log_2$(counts per million + 1) expression (computed manually) for each spatial domain (*x*-axis) in the pseudobulked dlPFC SRT data. Color represents the spatial domain. Fourth column (**D, H, L, P**): Spot plot of dlPFC Visium capture area from donor Br6432 (sample ID: Br6432_ant) with spots colored by the dlPFC spatial domains. Fill represents $\log_2$-normalized expression per spot.

bioRxiv preprint doi: https://doi.org/10.1101/2025.07.14.664821; this version posted July 17, 2025. The copyright holder for this preprint (which was not certified by peer review) is the author/funder, who has granted bioRxiv a license to display the preprint in perpetuity. It is made available under aCC-BY 4.0 International license.

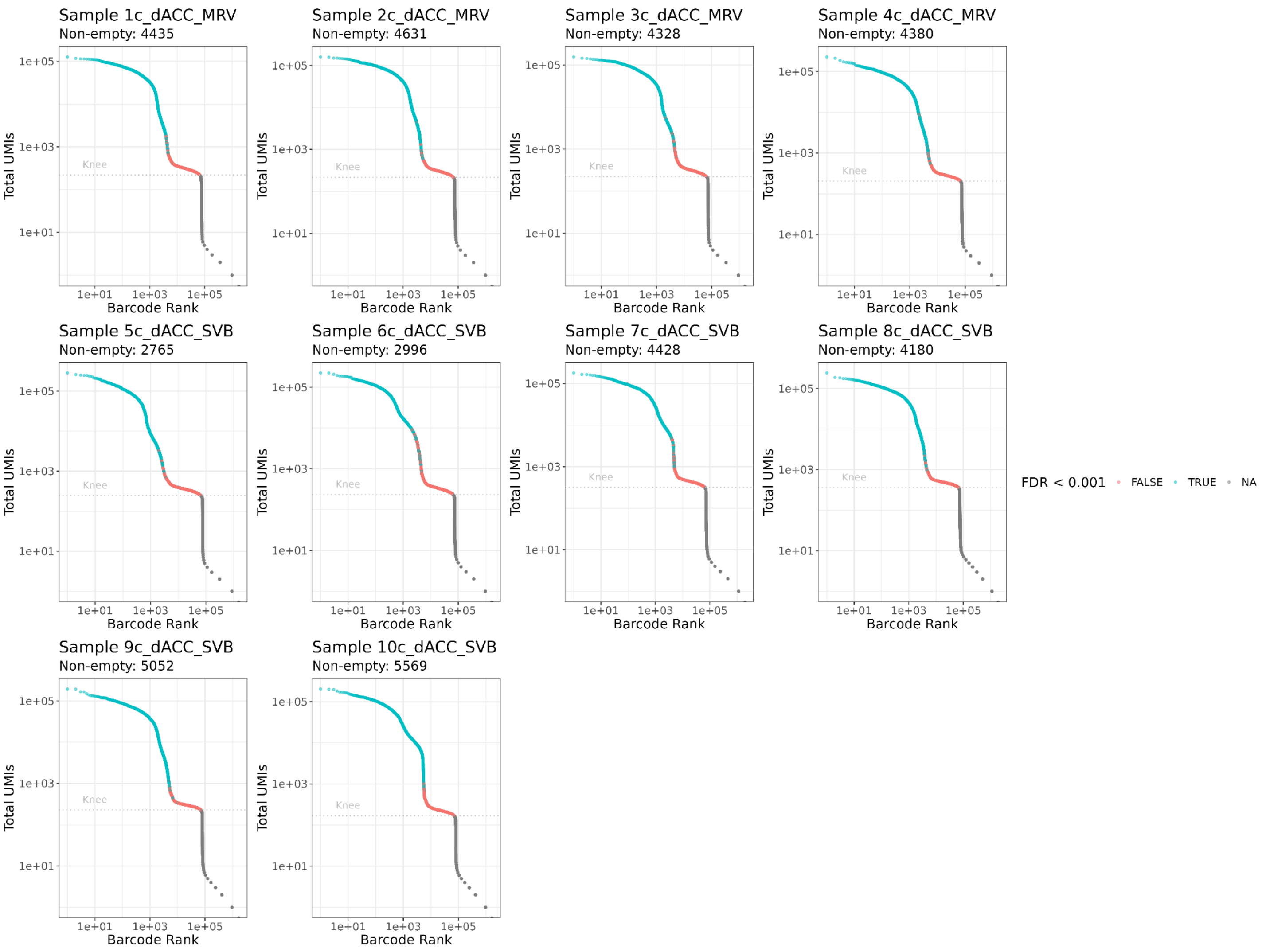

Supplementary Fig. 14. Empty droplets preprocessing of dACC snRNA-seq data. `DropletUtils` knee plots identify sample-specific UMI thresholds for empty droplets preprocessing in the dACC snRNA-seq data. Color represents quality based on an FDR cutoff of 0.001: blue indicates predicted real nuclei with FDR < 0.001 and red indicates predicted empty nuclei with FDR > 0.001. The horizontal dotted line represents the knee point used as an empty droplet cutoff. Each knee plot represents a different sample.

bioRxiv preprint doi: https://doi.org/10.1101/2025.07.14.664821; this version posted July 17, 2025. The copyright holder for this preprint (which was not certified by peer review) is the author/funder, who has granted bioRxiv a license to display the preprint in perpetuity. It is made available under aCC-BY 4.0 International license.

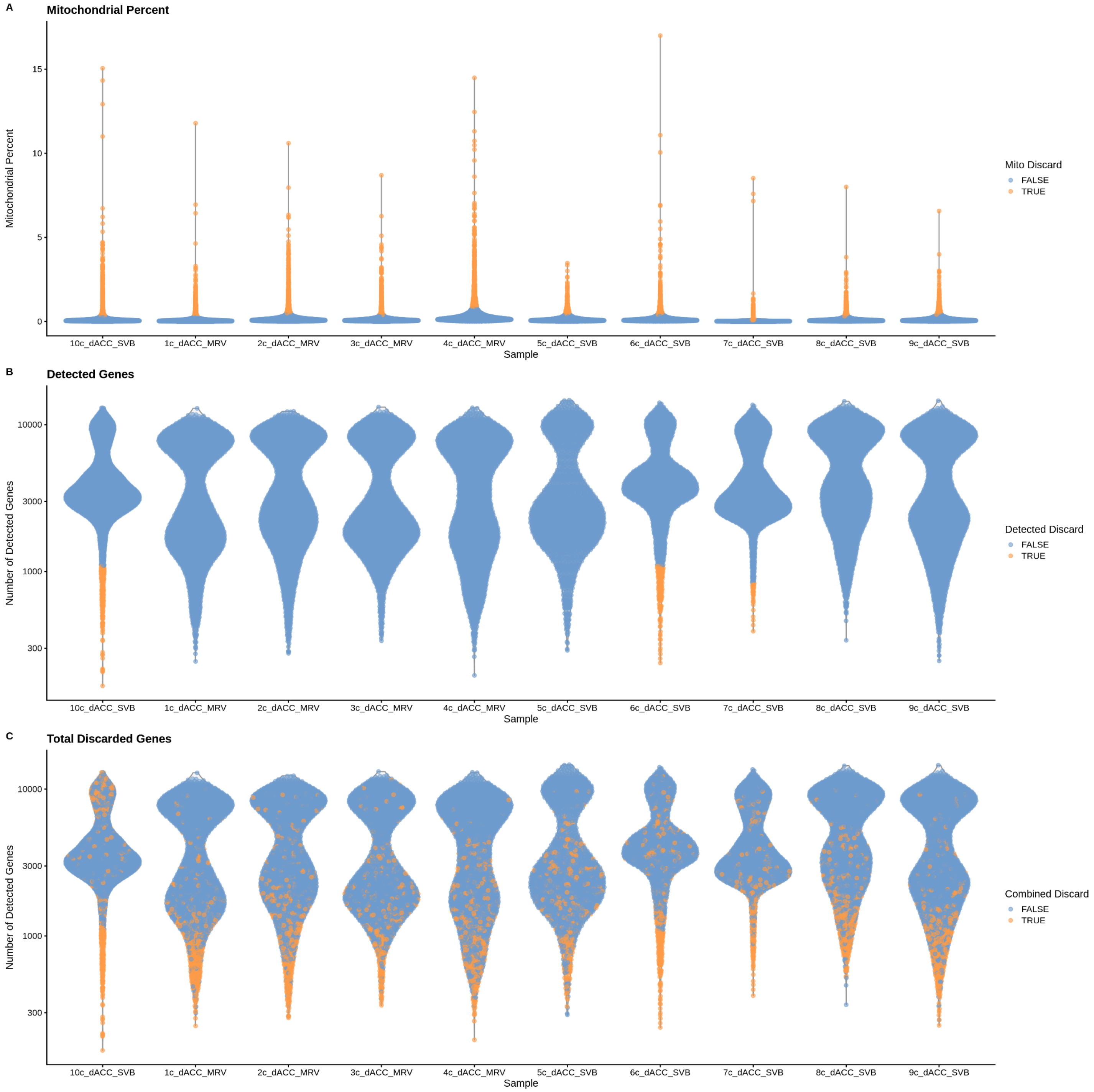

Supplementary Fig. 15. **Quality control (QC) metrics for dACC snRNA-seq data.** Color represents discarded status: yellow indicates nuclei that were dropped, while blue indicates nuclei used for downstream analyses. (**A**) Violin plot shows the mitochondrial percentage (*y*-axis) for each nucleus, separately plotted for each sample (*x*-axis). (**B**) Violin plot shows the number of detected genes (*y*-axis) for each nucleus, separately plotted for each sample (*x*-axis). (**C**) Violin plot shows the number of detected genes (*y*-axis) for each nucleus, separately plotted for each sample (*x*-axis). The color represents nuclei discarded based on mitochondrial percentage and number of detected genes. Thresholds were determined with `scuttle` (McCarthy et al. 2017).

bioRxiv preprint doi: https://doi.org/10.1101/2025.07.14.664821; this version posted July 17, 2025. The copyright holder for this preprint (which was not certified by peer review) is the author/funder, who has granted bioRxiv a license to display the preprint in perpetuity. It is made available under aCC-BY 4.0 International license.

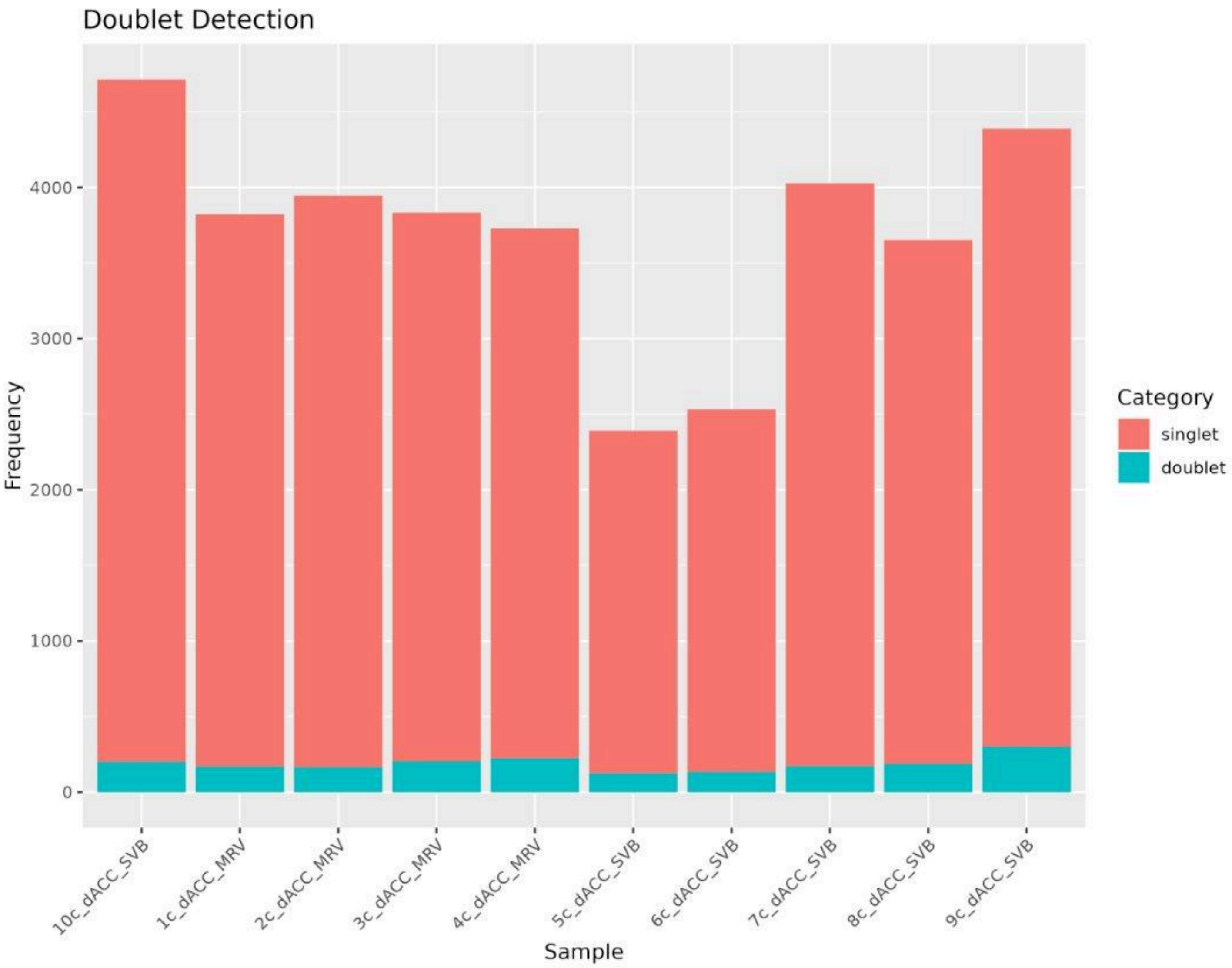

Supplementary Fig. 16. **Doublet detection removes a small proportion of nuclei in dACC snRNA-seq data.** Barplots quantify the frequency (*y*-axis) of doublets and singlets in each sample (*x*-axis) of the dACC snRNA-seq data. Color represents the proportion of singlets and doublets in each sample. Doublets were detected with `scDblFinder (Germain et al. 2021).`

bioRxiv preprint doi: https://doi.org/10.1101/2025.07.14.664821; this version posted July 17, 2025. The copyright holder for this preprint (which was not certified by peer review) is the author/funder, who has granted bioRxiv a license to display the preprint in perpetuity. It is made available under aCC-BY 4.0 International license.

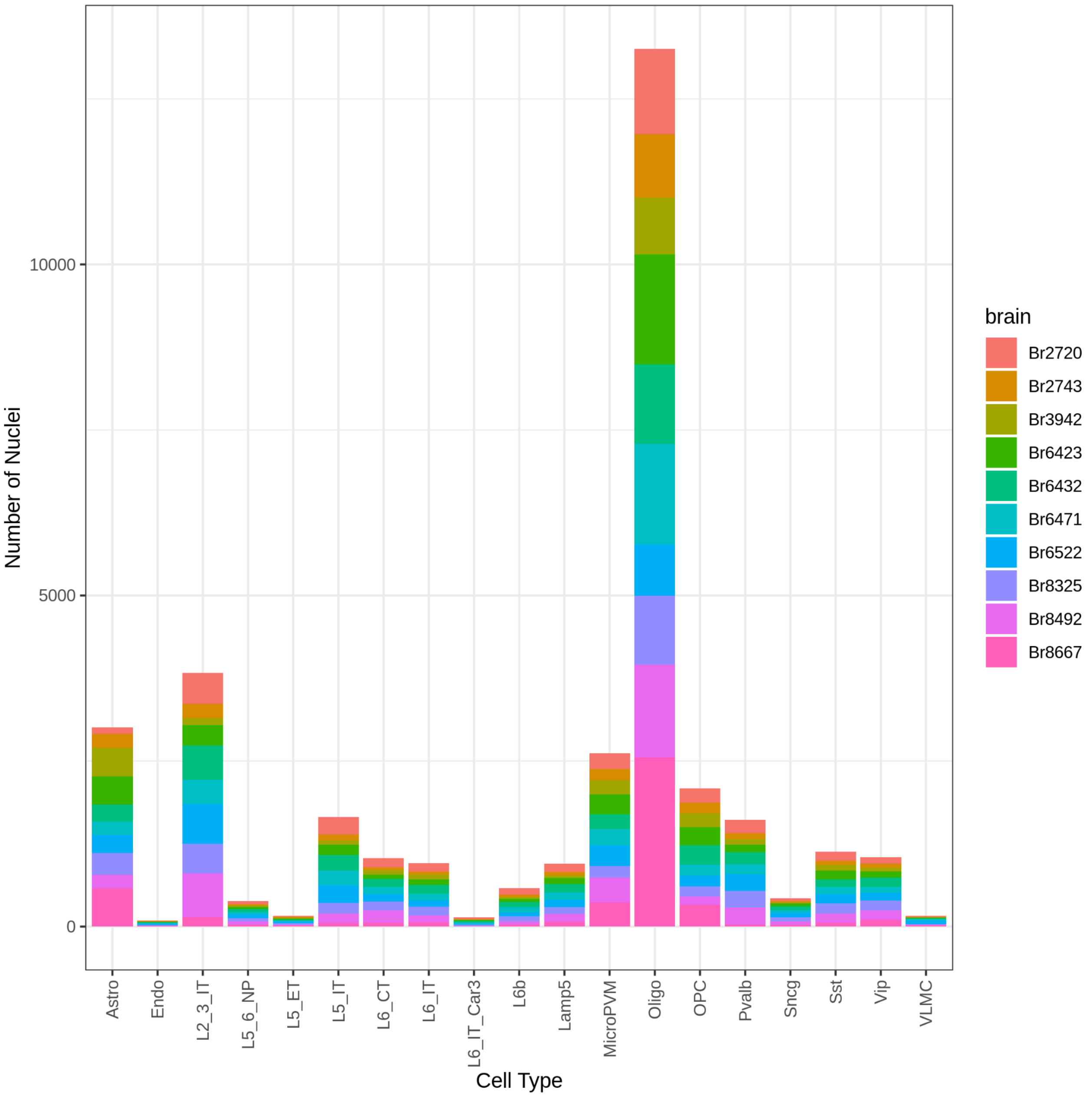

Supplementary Fig. 17. **Brain donor composition of dACC snRNA-seq cell types.** Barplot displays the number of nuclei (*y*-axis) in each cell type (*x*-axis) in the dACC snRNA-seq data. Color represents the proportion of nuclei in each cell type coming from each donor.

bioRxiv preprint doi: https://doi.org/10.1101/2025.07.14.664821; this version posted July 17, 2025. The copyright holder for this preprint (which was not certified by peer review) is the author/funder, who has granted bioRxiv a license to display the preprint in perpetuity. It is made available under aCC-BY 4.0 International license.

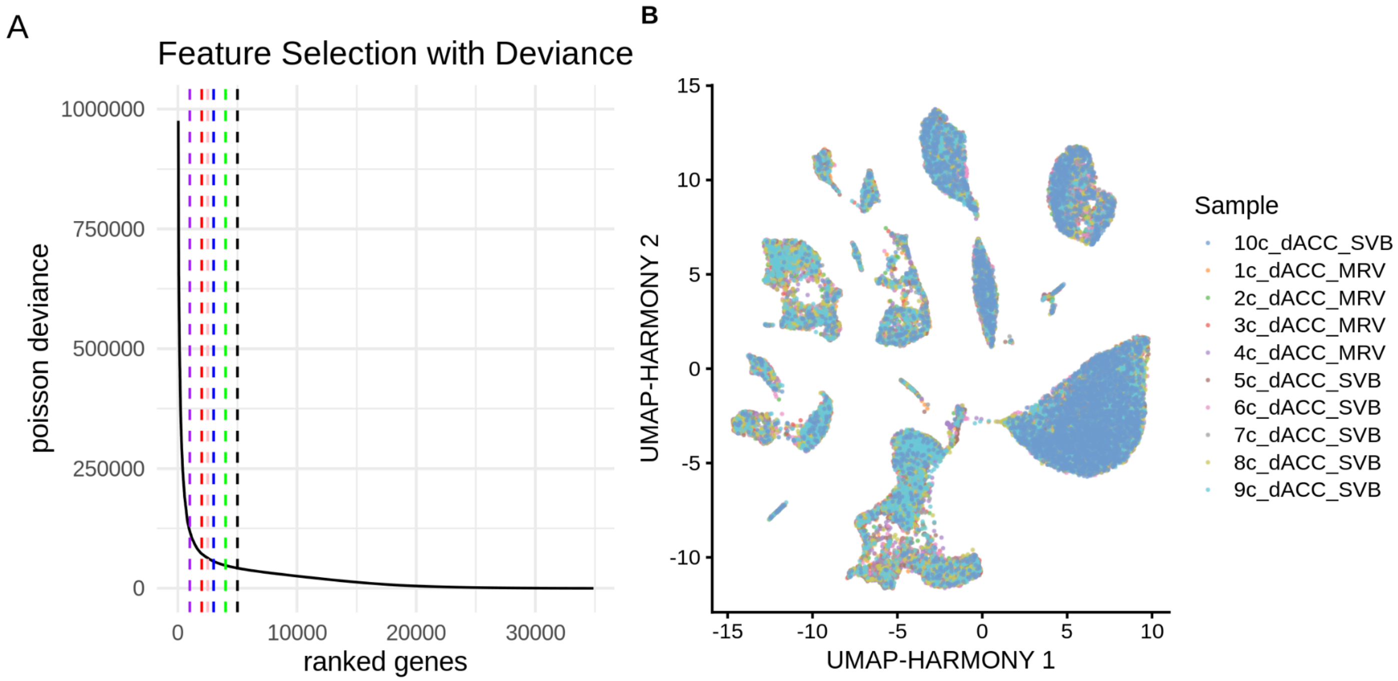

Supplementary Fig. 18. **Dimension reduction of dACC snRNA-seq data.** (**A**) The line plot shows a deviance feature selection intermediate figure. The Poisson deviance (*y*-axis) was calculated using the dACC snRNA-seq counts matrix and is plotted against the number of ranked genes (*x*-axis). The dashed lines mark 1,000, 2,000, 2,500, 3,000, 4,000, and 5,000 genes. (**B**) Uniform manifold approximation and projection (UMAP) representation of the dACC snRNA-seq dataset after Harmony batch correction. Color represents the sample for each nucleus.

bioRxiv preprint doi: https://doi.org/10.1101/2025.07.14.664821; this version posted July 17, 2025. The copyright holder for this preprint (which was not certified by peer review) is the author/funder, who has granted bioRxiv a license to display the preprint in perpetuity. It is made available under aCC-BY 4.0 International license.

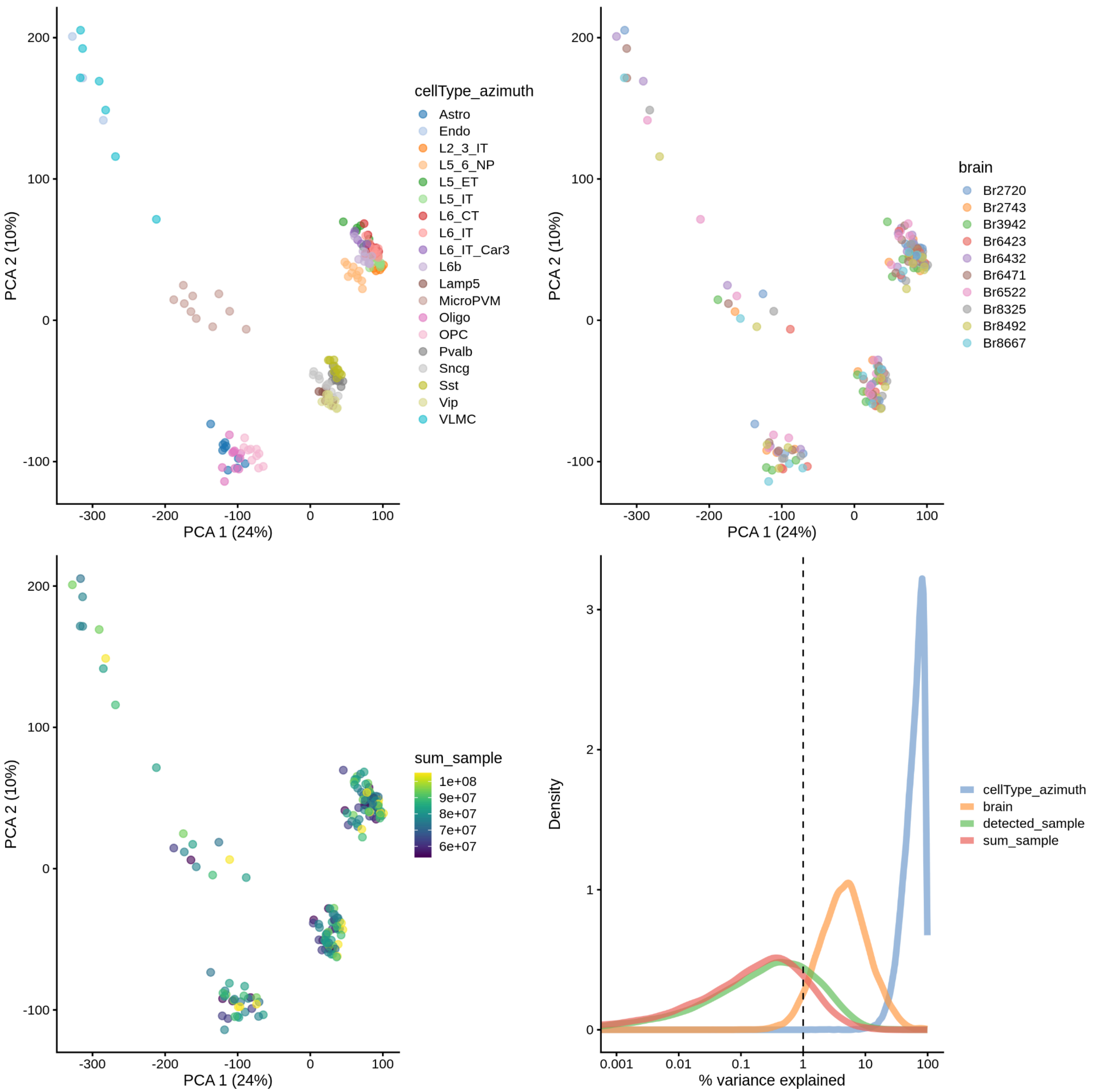

Supplementary Fig. 19. **Principal components analysis (PCA) of pseudobulked dACC snRNA-seq data.** The first three plots show the score plots of the first two (PCs) of the pseudobulked dACC snRNA-seq cell types. Each score plot is colored by cell type, brain donor, and total UMI counts per sample, respectively. The fourth plot, made with `scater (McCarthy et al. 2017)`, shows the percent variance explained by cell type, brain donor, total UMI counts per sample, and total detected genes per sample.

bioRxiv preprint doi: https://doi.org/10.1101/2025.07.14.664821; this version posted July 17, 2025. The copyright holder for this preprint (which was not certified by peer review) is the author/funder, who has granted bioRxiv a license to display the preprint in perpetuity. It is made available under aCC-BY 4.0 International license.

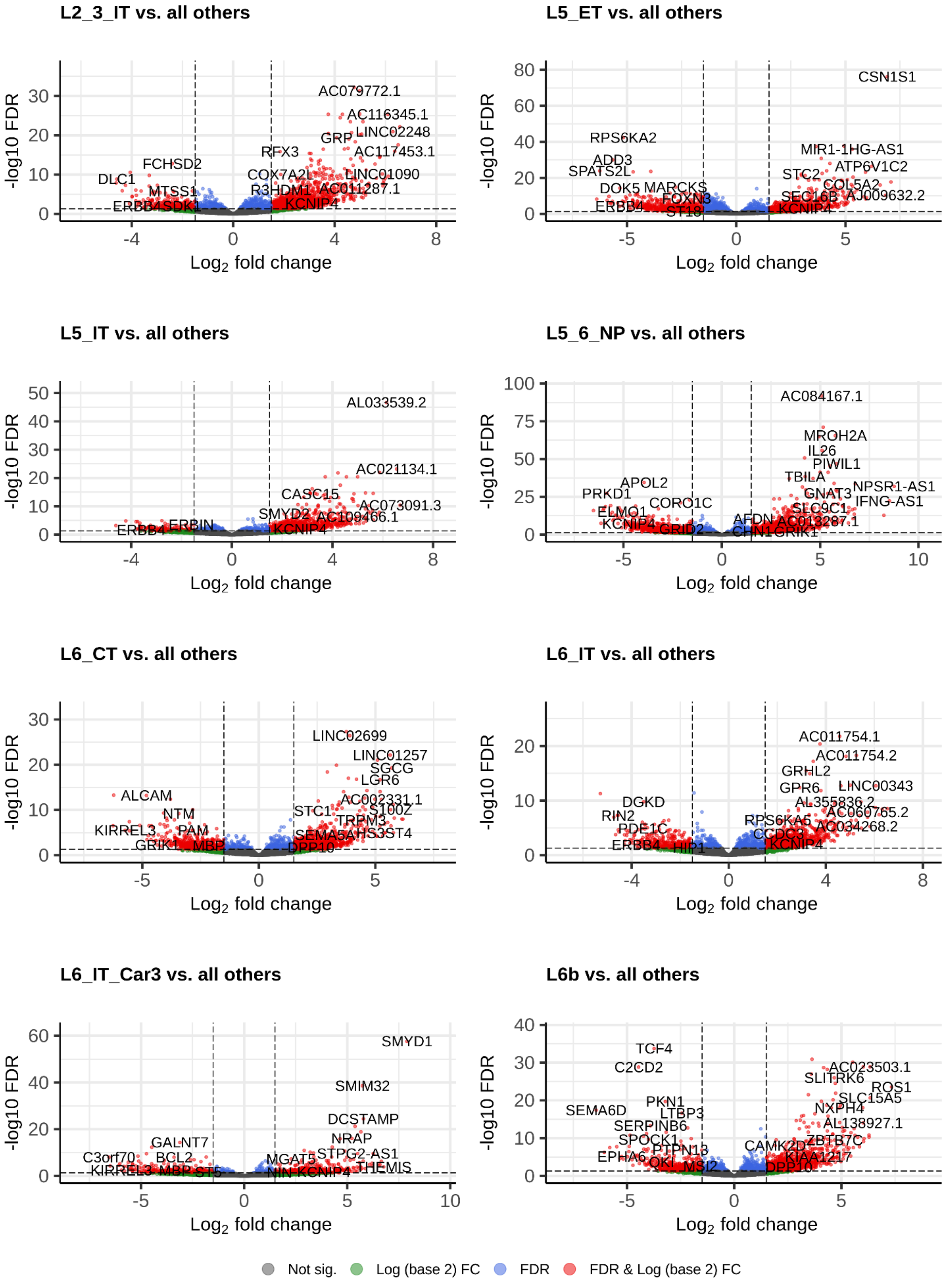

Supplementary Fig. 20. Differential expression (DE) analysis of pseudobulked dACC snRNA-seq layer-specific cell types. Each `EnhancedVolcano` plot shows the DE results for the enrichment model pseudobulked test for one dACC snRNA-seq cell type compared to all other dACC snRNA-seq cell types. Each

bioRxiv preprint doi: https://doi.org/10.1101/2025.07.14.664821; this version posted July 17, 2025. The copyright holder for this preprint (which was not certified by peer review) is the author/funder, who has granted bioRxiv a license to display the preprint in perpetuity. It is made available under aCC-BY 4.0 International license.

point is a gene with its log fold-change (logFC) (*x*-axis) and statistical significance (*y*-axis). Statistical significance is measured with negative log-transformation of FDR-adjusted *p*-values. Color indicates categorization of each gene; red represents statistically significant with FDR < 0.05 and absolute value of logFC > 1, blue represents statistically significant with FDR < 0.05 only, and grey represents not statistically significant with FDR >= 0.05. These plots are a subset of only the layer-specific cell types in the dACC snRNA-seq data.

bioRxiv preprint doi: https://doi.org/10.1101/2025.07.14.664821; this version posted July 17, 2025. The copyright holder for this preprint (which was not certified by peer review) is the author/funder, who has granted bioRxiv a license to display the preprint in perpetuity. It is made available under aCC-BY 4.0 International license.

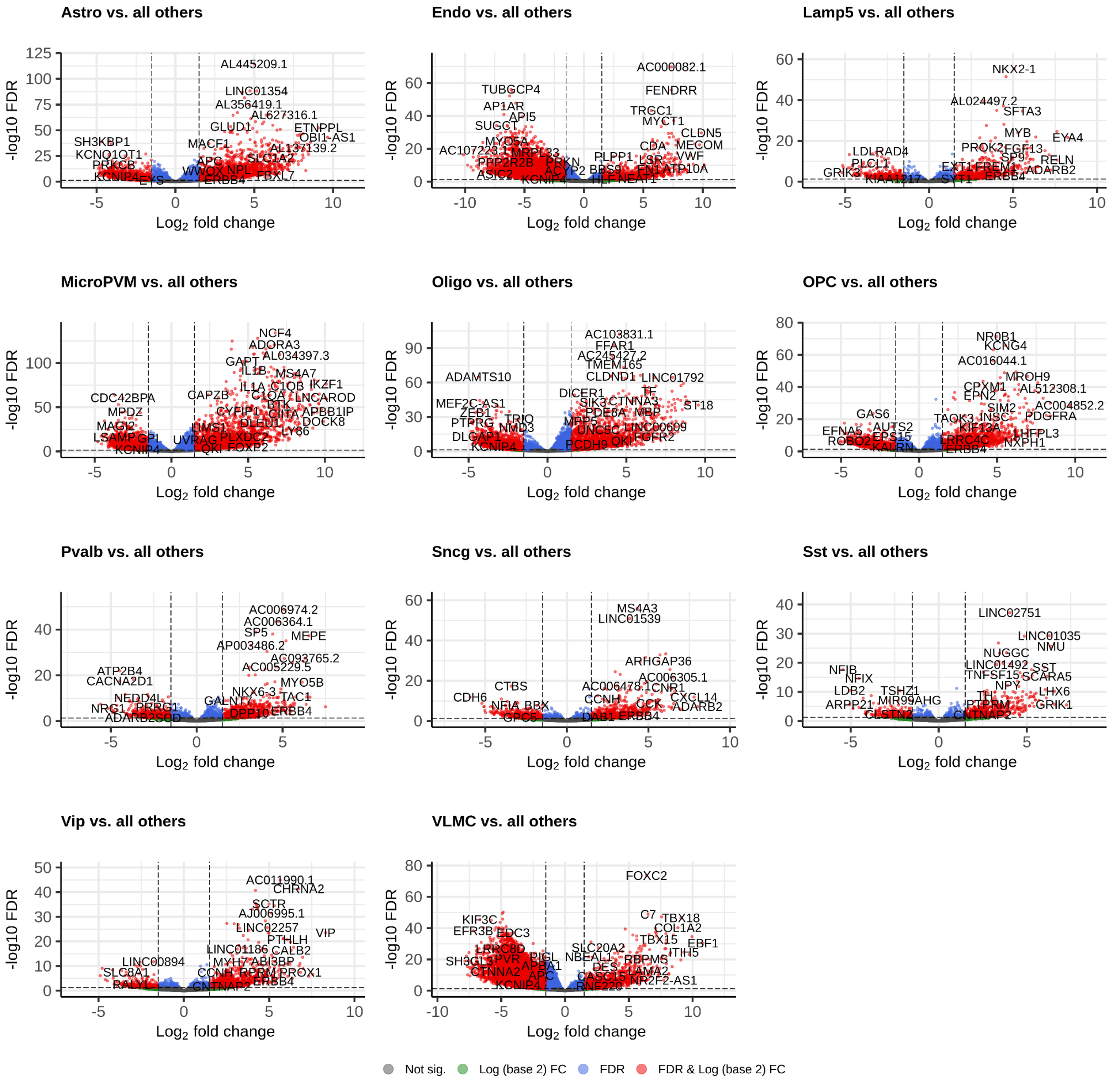

Supplementary Fig. 21. **Differential expression (DE) analysis of pseudobulked dACC snRNA-seq non-layer-specific cell types.** Each `EnhancedVolcano` plot shows the DE results for the enrichment model pseudobulked test for one dACC snRNA-seq cell type compared to all other dACC snRNA-seq cell types. Each point is a gene with its log fold-change (logFC) (*x*-axis) and statistical significance (*y*-axis). Statistical significance is measured with negative log-transformation of FDR-adjusted *p*-values. Color indicates categorization of each gene; red represents statistically significant with FDR < 0.05 and absolute value of logFC > 1, blue represents statistically significant with FDR < 0.05 only, and grey represents not statistically significant with FDR >= 0.05. These plots are a subset of only the non-layer-specific cell types in the dACC snRNA-seq data.

bioRxiv preprint doi: https://doi.org/10.1101/2025.07.14.664821; this version posted July 17, 2025. The copyright holder for this preprint (which was not certified by peer review) is the author/funder, who has granted bioRxiv a license to display the preprint in perpetuity. It is made available under aCC-BY 4.0 International license.

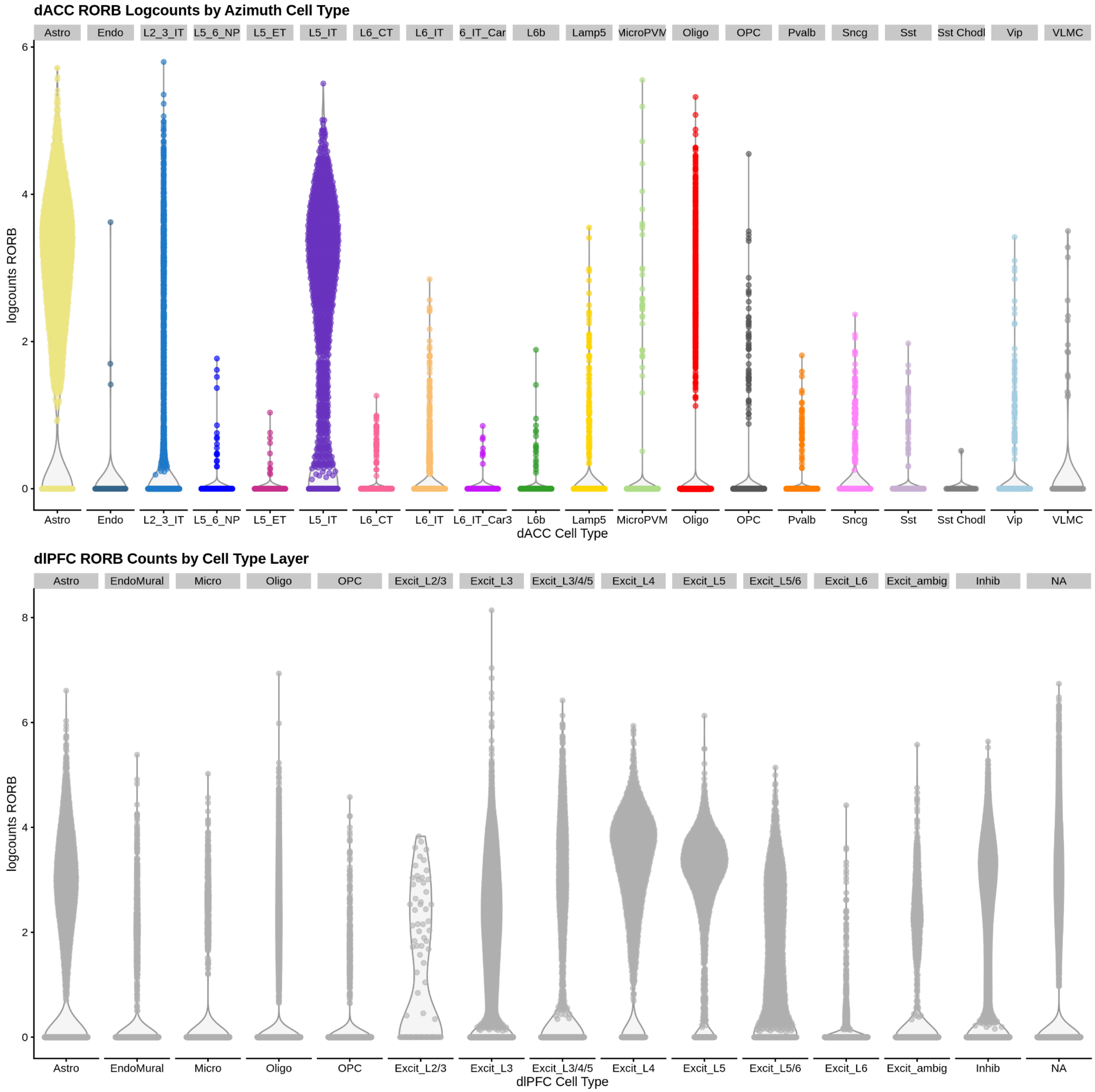

Supplementary Fig. 22. **Expression of *RORB* in dlPFC and dACC snRNA-seq data.** Top violin plots show $\log_2$-normalized expression (*y*-axis) of *RORB* across the `Azimuth` cell types (*x*-axis) in the dACC snRNA-seq data. Color represents cell type. Bottom violin plots show $\log_2$-normalized expression (*y*-axis) of *RORB* across the cell type layer annotations (*x*-axis) in the dlPFC snRNA-seq data.

bioRxiv preprint doi: https://doi.org/10.1101/2025.07.14.664821; this version posted July 17, 2025. The copyright holder for this preprint (which was not certified by peer review) is the author/funder, who has granted bioRxiv a license to display the preprint in perpetuity. It is made available under aCC-BY 4.0 International license.

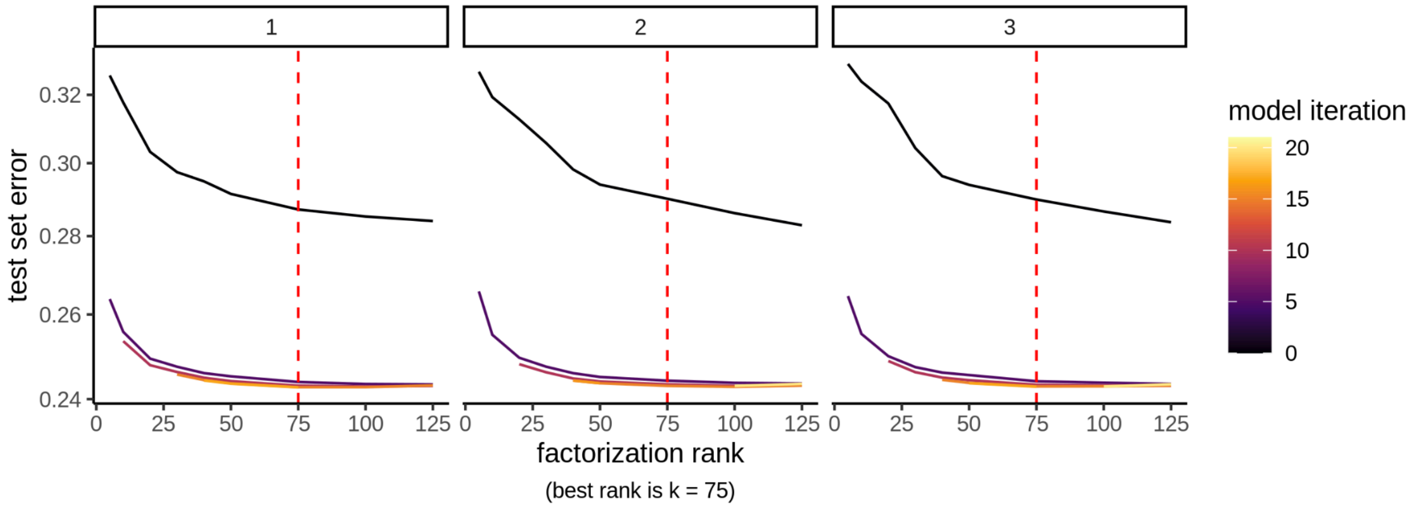

Supplementary Fig. 23. **Non-negative matrix factorization (NMF) cross-validation on dACC snRNA-seq data indicates *k*=75 as optimal rank.** Each line plot shows 1 replicate of NMF cross-validation on the $\log_2$-normalized dACC snRNA-seq data, which was computed on the ranks of 5, 10, 20, 30, 40, 50, 75, 100, and 125 (*x*-axis). The test set error is plotted on the *y*-axis. The cross-validation software `singlet` identified *k*=75 as the optimal rank to use for NMF factorization.

bioRxiv preprint doi: https://doi.org/10.1101/2025.07.14.664821; this version posted July 17, 2025. The copyright holder for this preprint (which was not certified by peer review) is the author/funder, who has granted bioRxiv a license to display the preprint in perpetuity. It is made available under aCC-BY 4.0 International license.

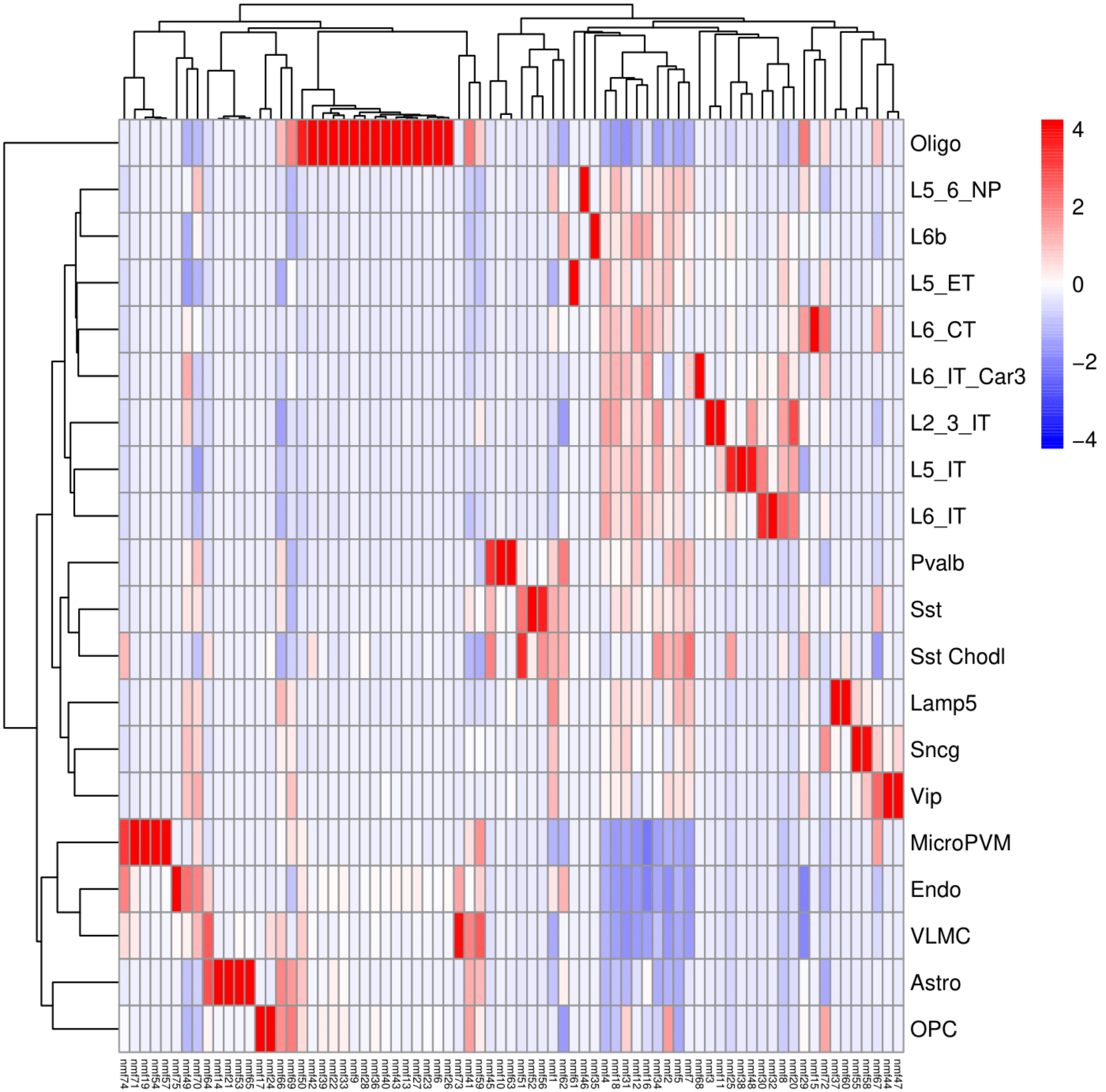

Supplementary Fig. 24. **Non-negative matrix factorization (NMF) patterns of dACC snRNA-seq data associated with snRNA-seq cell types.** Heatmap displays the correlation between the 75 NMF patterns from the dACC snRNA-seq data (y-axis) and snRNA-seq cell types (x-axis). The NMF patterns were aggregated across cell types, and the mean of each NMF pattern within each cell type is displayed.

bioRxiv preprint doi: https://doi.org/10.1101/2025.07.14.664821; this version posted July 17, 2025. The copyright holder for this preprint (which was not certified by peer review) is the author/funder, who has granted bioRxiv a license to display the preprint in perpetuity. It is made available under aCC-BY 4.0 International license.

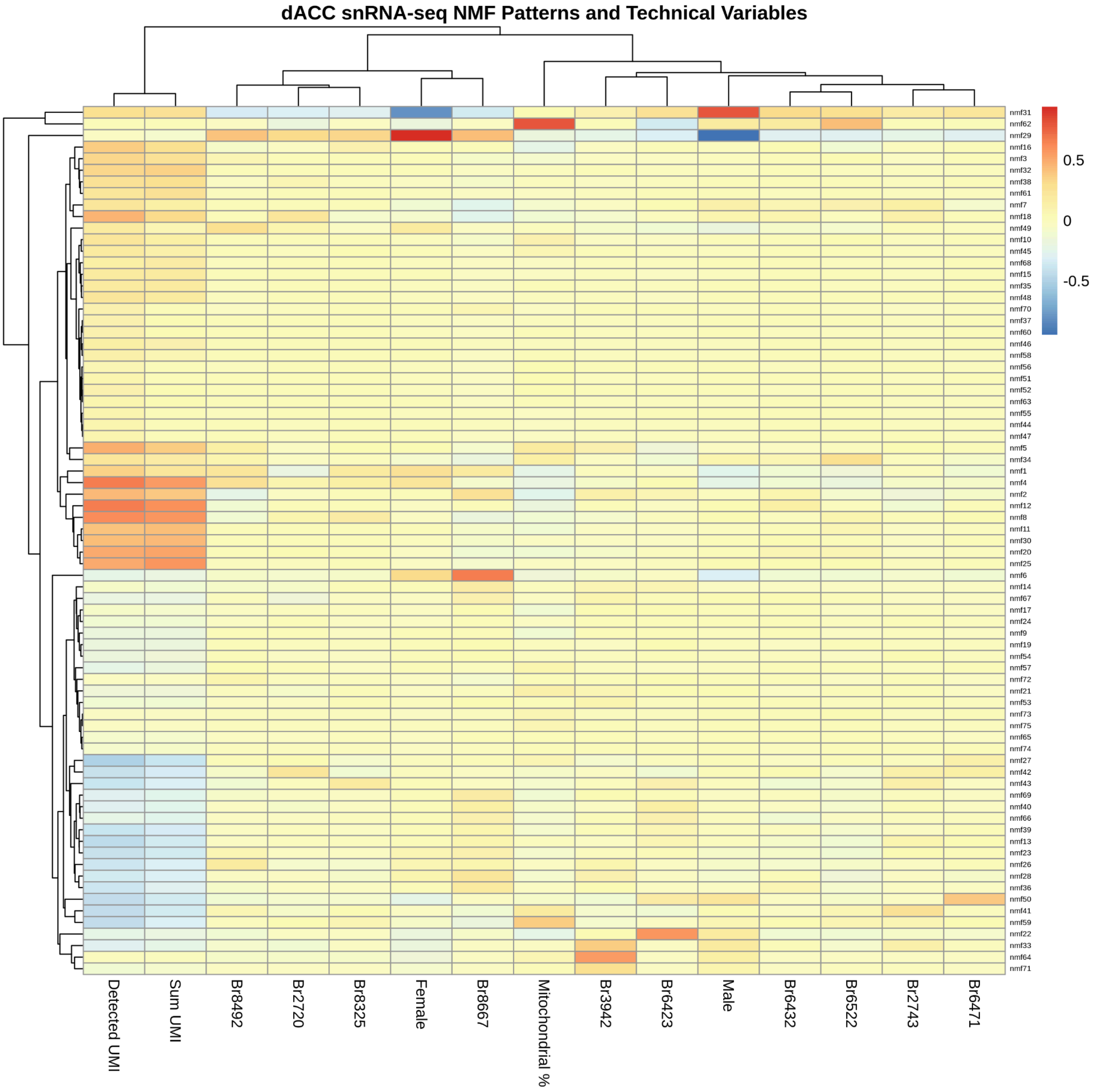

Supplementary Fig. 25. **Non-negative matrix factorization (NMF) patterns of dACC snRNA-seq data associated with technical variables.** Heatmap displays the correlation between the 75 NMF patterns from the dACC snRNA-seq data (*y*-axis) and technical variables related to brain donor, sex of brain donor (Male or Female), and quality control metrics (Detected UMI counts, Sum UMI counts, and Mitochondrial Percentage) (*x*-axis).

bioRxiv preprint doi: https://doi.org/10.1101/2025.07.14.664821; this version posted July 17, 2025. The copyright holder for this preprint (which was not certified by peer review) is the author/funder, who has granted bioRxiv a license to display the preprint in perpetuity. It is made available under aCC-BY 4.0 International license.

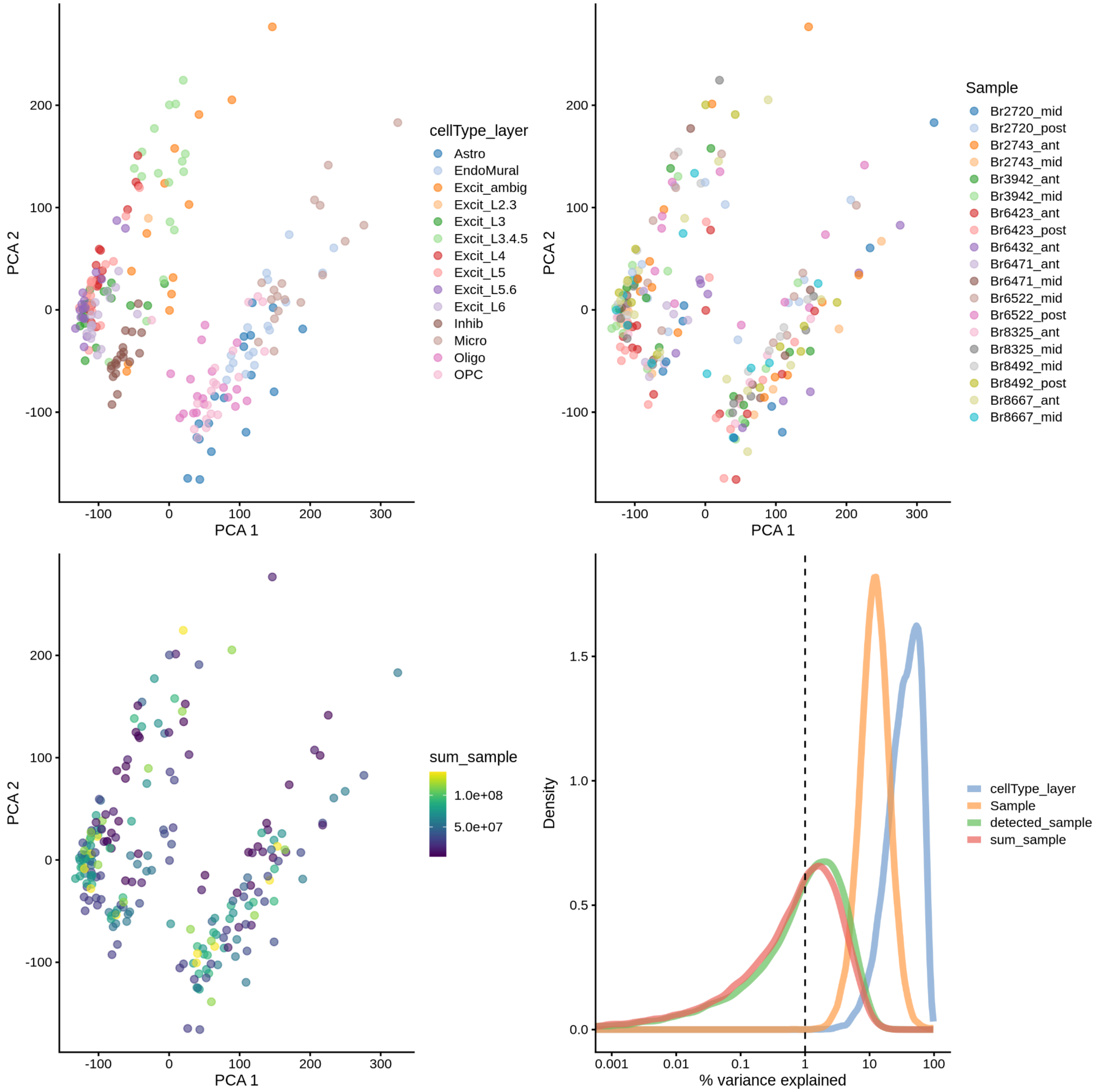

Supplementary Fig. 26. **Principal components analysis (PCA) of pseudobulked dlPFC snRNA-seq data.** The first three plots show the score plots of the first two (PCs) of the pseudobulked dlPFC snRNA-seq cell types. Each score plot is colored by cell type, sample id, and total UMI counts per sample, respectively. The fourth plot, made with `scater (McCarthy et al. 2017)`, shows the percent variance explained by cell type, sample id, total UMI counts per sample, and total detected genes per sample.

bioRxiv preprint doi: https://doi.org/10.1101/2025.07.14.664821; this version posted July 17, 2025. The copyright holder for this preprint (which was not certified by peer review) is the author/funder, who has granted bioRxiv a license to display the preprint in perpetuity. It is made available under aCC-BY 4.0 International license.

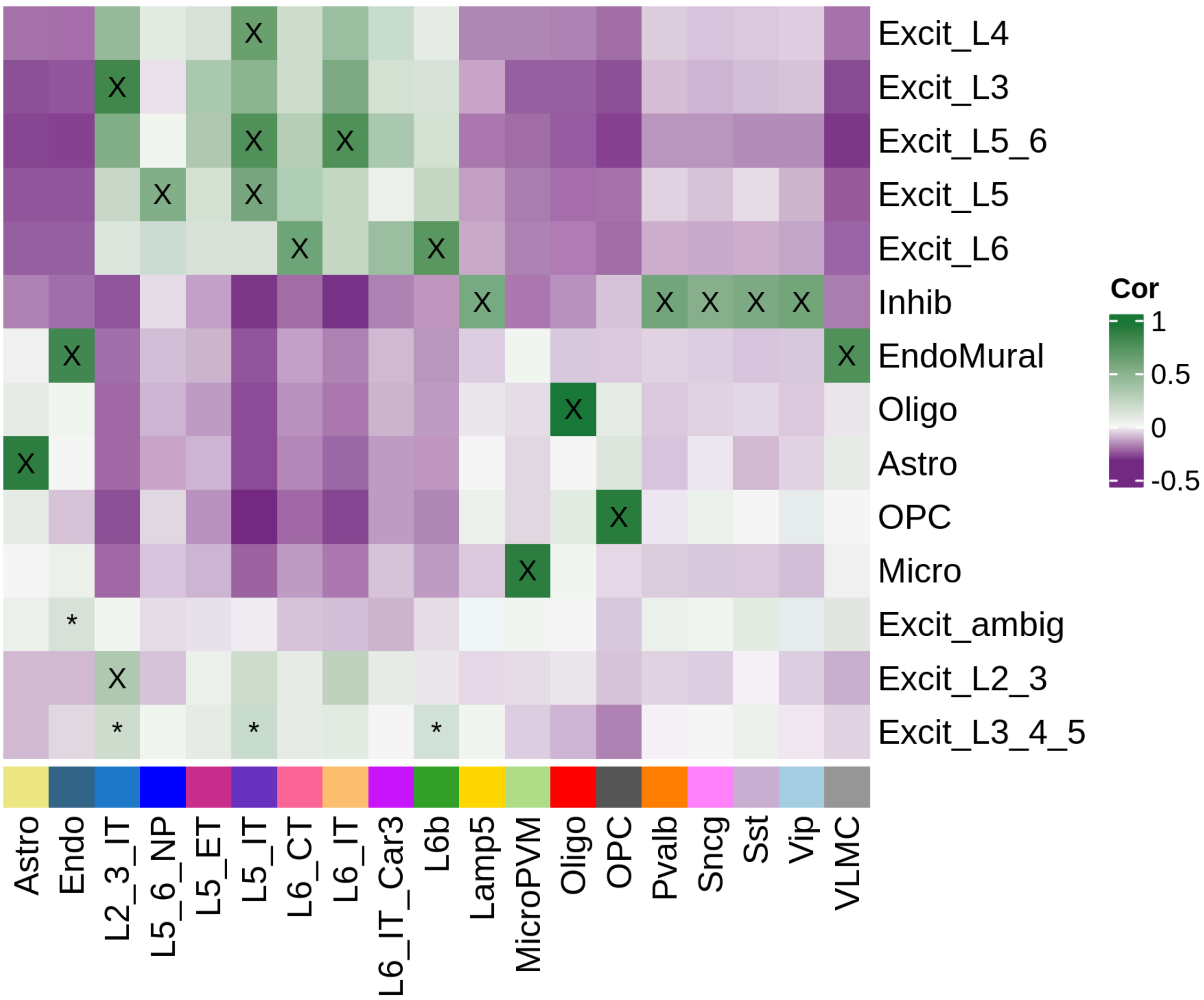

Supplementary Fig. 27. `spatialLIBD` spatial registration heatmap displays Pearson's correlation values between the top 100 marker genes in each dlPFC snRNA-seq cell type and in each dACC snRNA-seq cell type. The *x*-axis displays the dACC snRNA-seq cell types described in (**Figure 3A**). The *y*-axis displays dlPFC snRNA-seq cell types: Astro: Astrocyte; EndoMural: endothelial and mural cells; Excit L2/3: Layer 2/3 excitatory neurons; Excit L3: Layer 3 excitatory neurons; Excit L3/4/5: Layer 3/4/5 excitatory neurons; Excit L4: Layer 4 excitatory neurons; Excit L5: Layer 5 excitatory neurons; Excit L5/6: Layer 5/6 excitatory neurons; Excit L6: Layer 6 excitatory neurons; Excit ambig: ambiguous excitatory neurons; Micro: microglia; Inhib: inhibitory neurons; Oligo: oligodendrocytes; OPC: oligodendrocyte precursor cell. The black "X" represents high confidence and the black asterisk represents poor confidence (`confidence_threshold` = 0.25).

bioRxiv preprint doi: https://doi.org/10.1101/2025.07.14.664821; this version posted July 17, 2025. The copyright holder for this preprint (which was not certified by peer review) is the author/funder, who has granted bioRxiv a license to display the preprint in perpetuity. It is made available under aCC-BY 4.0 International license.

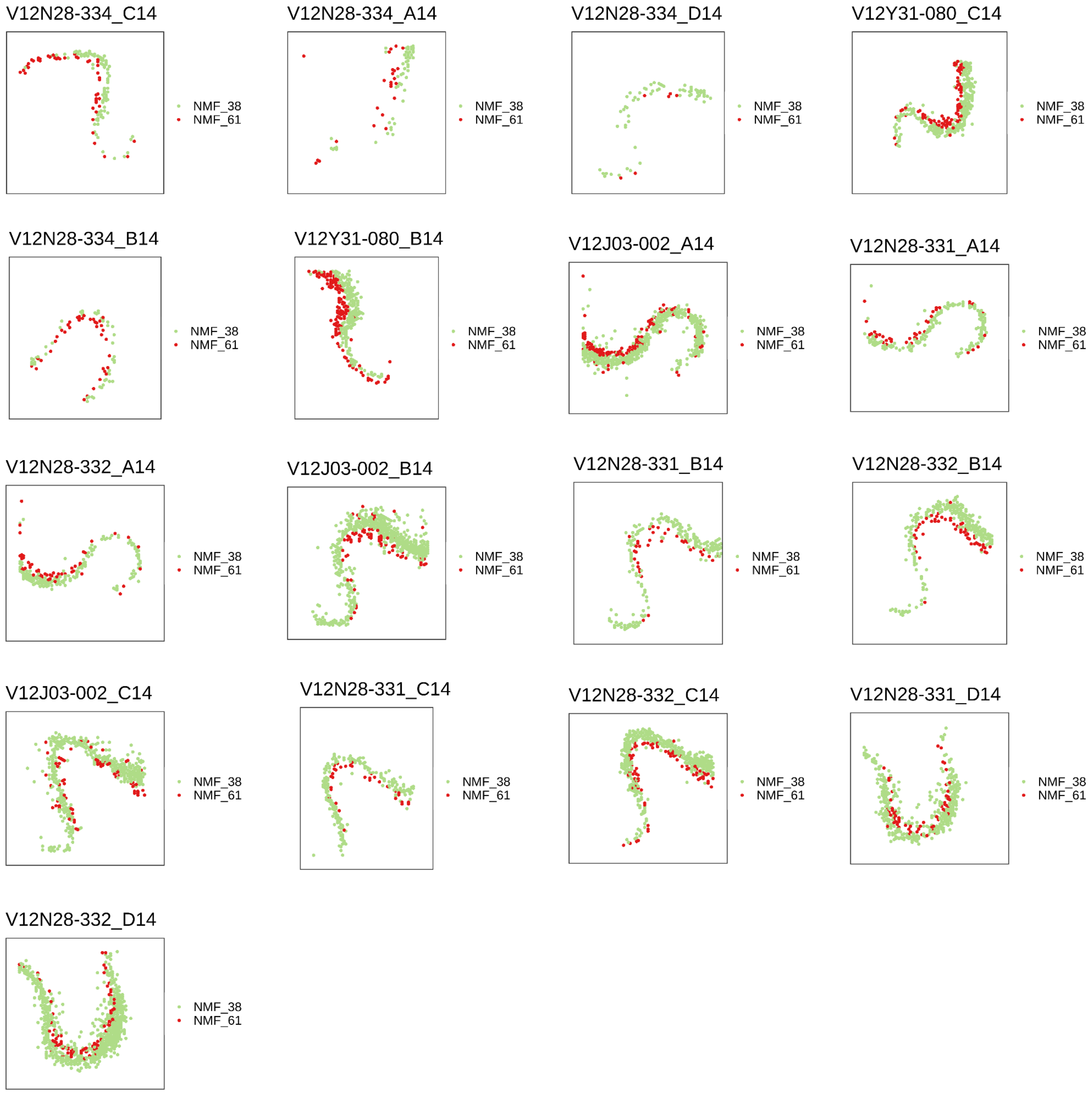

Supplementary Fig. 28. **Spot plot to display NMF patterns 38 and 61 mathematically projected into the dACC SRT data.** Each plot shows 1 sample of the dACC SRT data, with a subset of the spots from each sample that were predicted as either NMF38 or NMF61. Color represents spots that were predicted as either NMF38 (green) or NMF61 (red).

bioRxiv preprint doi: https://doi.org/10.1101/2025.07.14.664821; this version posted July 17, 2025. The copyright holder for this preprint (which was not certified by peer review) is the author/funder, who has granted bioRxiv a license to display the preprint in perpetuity. It is made available under aCC-BY 4.0 International license.

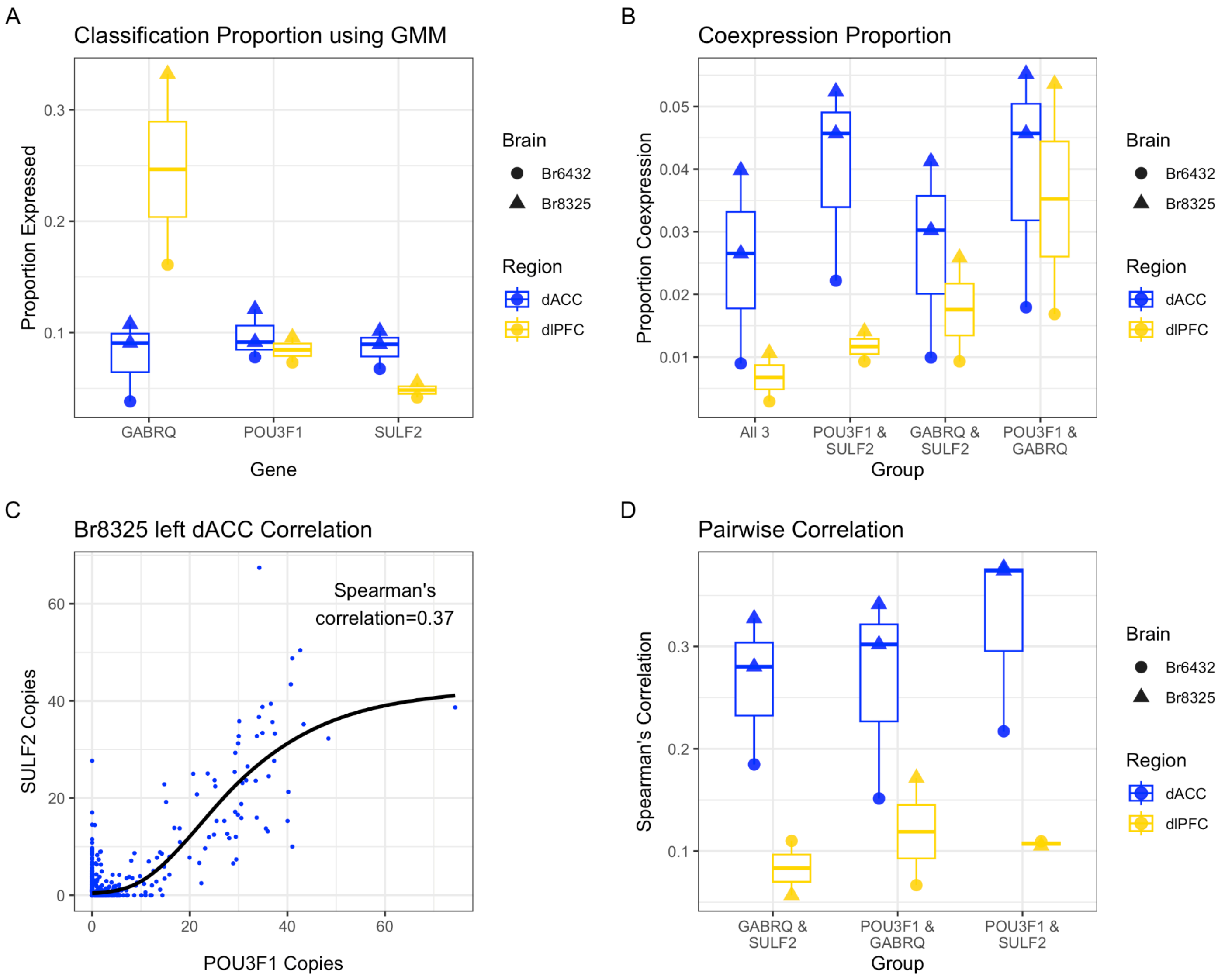

Supplementary Fig. 29. Analysis of RNAScope copy counts for VEN marker genes in dACC and dlPFC Layer 5 SRT data. (**A**) A Gaussian mixture model (GMM) with *k*=2 was used to classify the cells from each gene and sample as either expressing or not expressing the gene of interest. Boxplots display the proportion of cells (*y*-axis) from Layer 5 that were called as expressing each gene (*GABRQ*, *POU3F1*, and *SULF2*) (*x*-axis). Color indicates the region of the sample, either dACC or dlPFC. Shape indicates the brain donor of the sample, either Br6432 or Br8325. Note that there are two dACC samples from Br8325. (**B**) Boxplots display the proportion of cells (*y*-axis) from Layer 5 that were called as expressing two or three genes (*x*-axis). Color and shape same as (**A**). (**C**) Scatterplot displays the relationship between *POU3F1* copy count (*x*-axis) and *SULF2* copy count (*y*-axis) in dACC sample Br8325. A black loess smoothed line is fit to the data, and the Spearman's correlation is displayed in the upper right corner. (**D**) Boxplots display Spearman's correlation values (*y*-axis) for each pair of genes (*x*-axis). Color and shape same as (**A**).

bioRxiv preprint doi: https://doi.org/10.1101/2025.07.14.664821; this version posted July 17, 2025. The copyright holder for this preprint (which was not certified by peer review) is the author/funder, who has granted bioRxiv a license to display the preprint in perpetuity. It is made available under aCC-BY 4.0 International license.

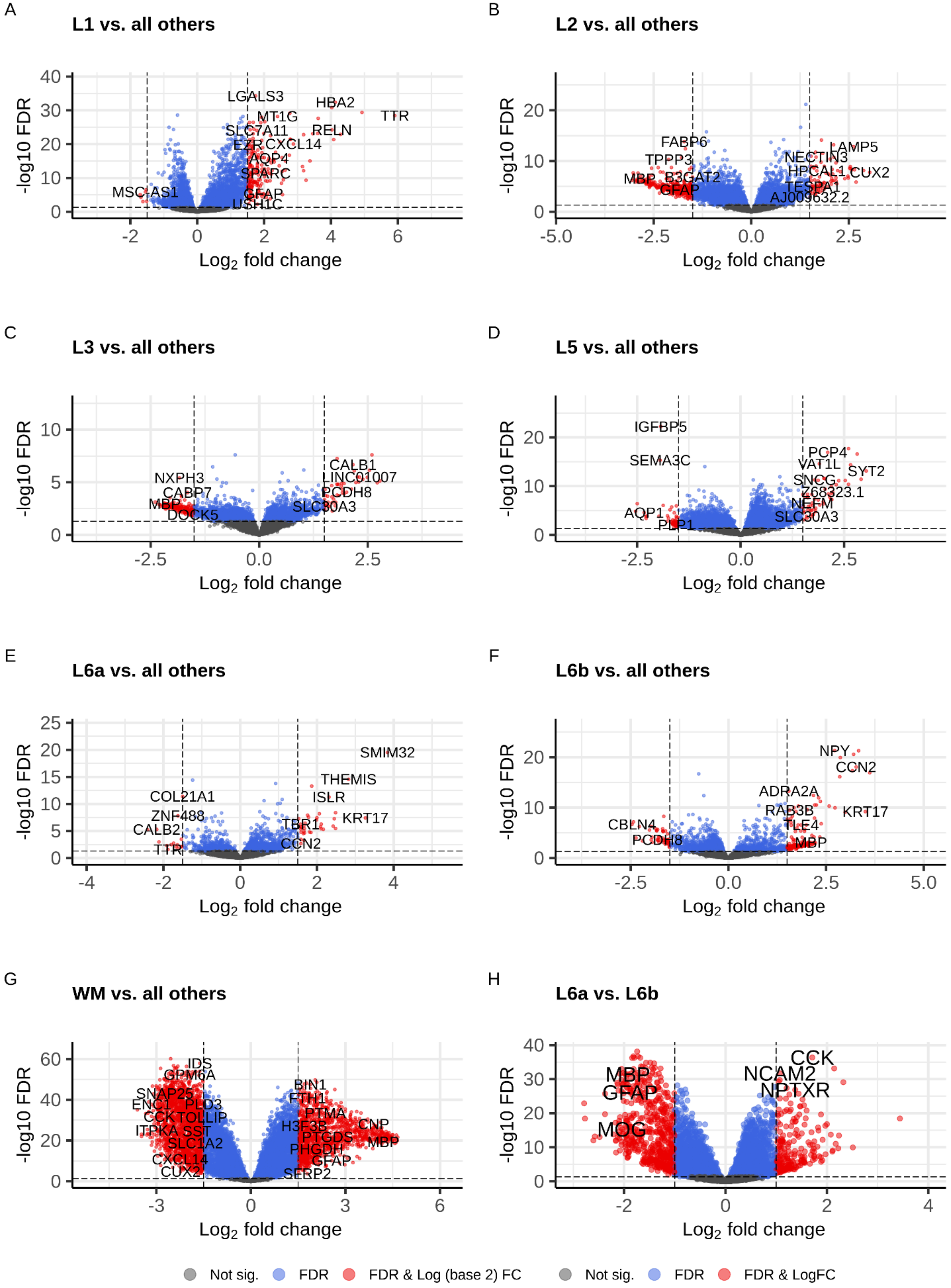

bioRxiv preprint doi: https://doi.org/10.1101/2025.07.14.664821; this version posted July 17, 2025. The copyright holder for this preprint (which was not certified by peer review) is the author/funder, who has granted bioRxiv a license to display the preprint in perpetuity. It is made available under aCC-BY 4.0 International license.

Supplementary Fig. 30. **Differential expression (DE) analysis of pseudobulked dACC SRT data. (A-G)** Each `EnhancedVolcano` plot shows the DE results for the enrichment model pseudobulked test for one dACC SRT spatial domain compared to all other dACC SRT spatial domains. Each point is a gene with its log fold-change (logFC) (*x*-axis) and statistical significance (*y*-axis). Statistical significance is measured with negative log-transformation of FDR-adjusted *p*-values. Color indicates categorization of each gene; red represents statistically significant with FDR < 0.05 and absolute value of logFC > 1, blue represents statistically significant with FDR < 0.05 only, and grey represents not statistically significant with FDR >= 0.05. **(H)** Similar to **(A-G)**, but the volcano plot shows DE results for the pairwise model pseudobulked test for dACC SRT spatial domain L6a compared to dACC SRT spatial domain L6b.

bioRxiv preprint doi: https://doi.org/10.1101/2025.07.14.664821; this version posted July 17, 2025. The copyright holder for this preprint (which was not certified by peer review) is the author/funder, who has granted bioRxiv a license to display the preprint in perpetuity. It is made available under aCC-BY 4.0 International license.

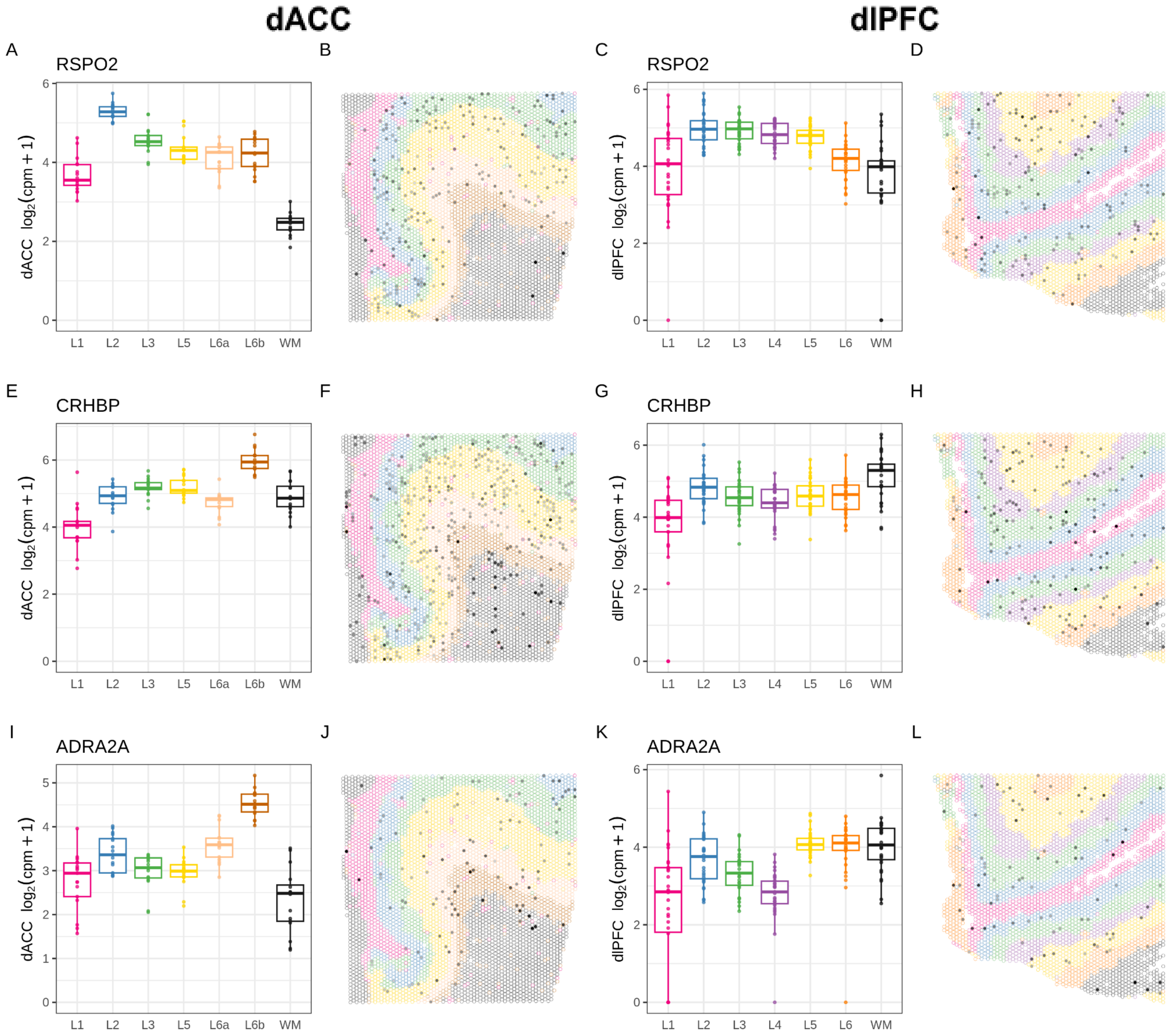

Supplementary Fig. 31. **Spatial domain layer markers in dACC and dlPFC SRT data.** Each row shows information for one gene, in order, *RSPO2, CRHBP, ADRA2A.* Each column displays a similar style of plot. First column (**A, E, I**): The *y*-axis displays $\log_2$(counts per million + 1) expression (computed manually) for each spatial domain (*x*-axis) in the pseudobulked dACC SRT data. Color represents the spatial domain. Second column (**B, F, J**): `escheR` spot plot of dACC Visium capture area from donor Br6432 (sample ID: V12N28-331_B1) with spots colored by the dACC spatial domains. Fill represents $\log_2$-normalized expression per spot. Third column (**C, G, K**): The *y*-axis displays $\log_2$(counts per million + 1) (computed manually) expression for each spatial domain (*x*-axis) in the pseudobulked dlPFC SRT data. Color represents the spatial domain. Fourth column (**D, H, L**): Spot plot of dlPFC Visium capture area from donor Br6432 (sample ID: Br6432_ant) with spots colored by the dlPFC spatial domains. Fill represents $\log_2$-normalized expression per spot.

bioRxiv preprint doi: https://doi.org/10.1101/2025.07.14.664821; this version posted July 17, 2025. The copyright holder for this preprint (which was not certified by peer review) is the author/funder, who has granted bioRxiv a license to display the preprint in perpetuity. It is made available under aCC-BY 4.0 International license.

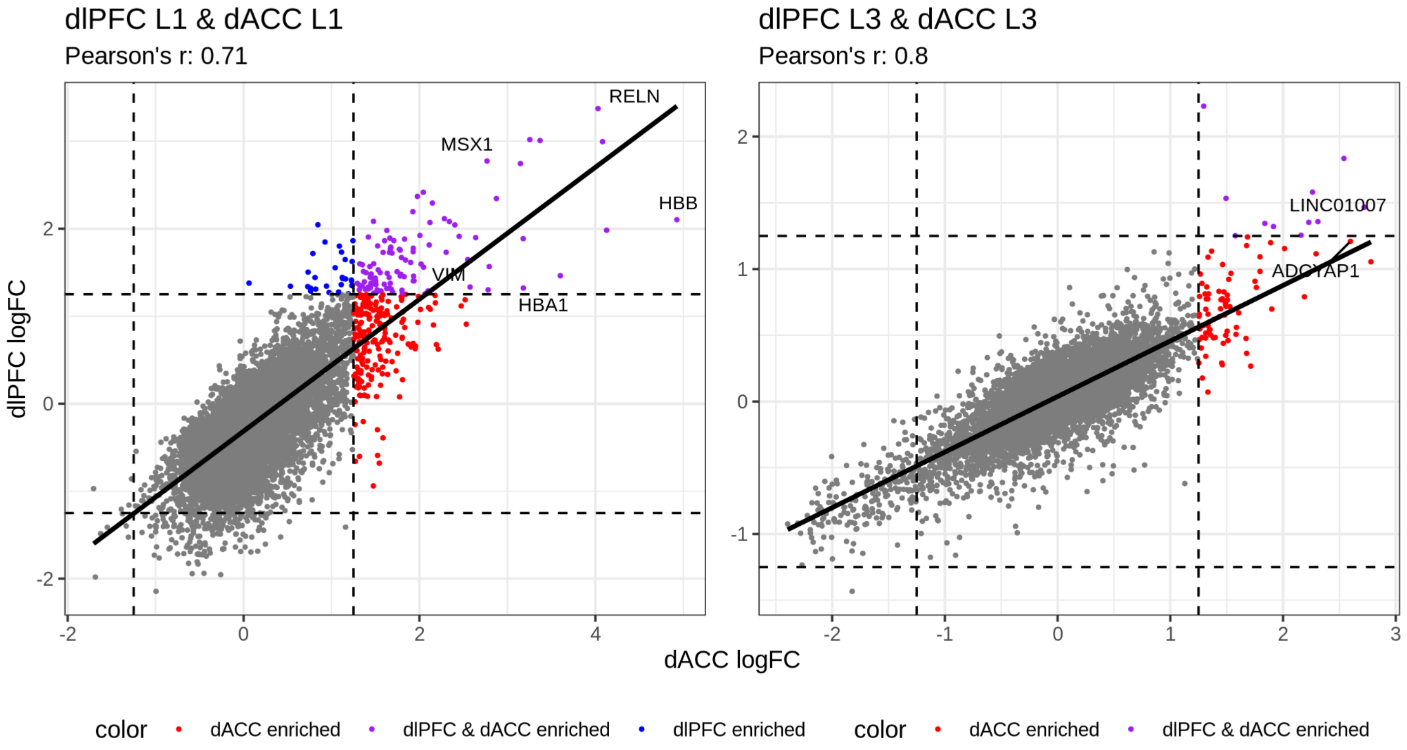

Supplementary Fig. 32. **Scatterplots comparing pseudobulked differential expression (DE) results per gene between dACC and dlPFC spatial domains.** Each scatterplot shows a comparison of the log fold-change (logFC) between one spatial domain in dACC and dlPFC (Huuki-Myers et al. 2024) SRT spatial domains. Each point is a gene. The *x*-axis of the first plot shows the logFC from enrichment model pseudobulked DE testing comparing Layer 1 to all other spatial domains for dACC, computing using `registration_wrapper()` (Pardo et al. 2022). The *y*-axis of the first plot shows the logFC from enrichment model pseudobulked DE testing comparing Layer 1 to all other spatial domains for dlPFC. The second plot compares L3 in the dACC to L3 in the dlPFC. The colors highlight genes that are classified as either dACC enriched (red), dlPFC enriched (blue), or both (purple), where the threshold is a logFC greater than 1.25. The number of genes in each category is as follows: L1) 197 dACC enriched & 28 dlPFC enriched & 101 dlPFC and dACC enriched and L3) 75 dACC enriched & 11 dlPFC and dACC enriched.

bioRxiv preprint doi: https://doi.org/10.1101/2025.07.14.664821; this version posted July 17, 2025. The copyright holder for this preprint (which was not certified by peer review) is the author/funder, who has granted bioRxiv a license to display the preprint in perpetuity. It is made available under aCC-BY 4.0 International license.

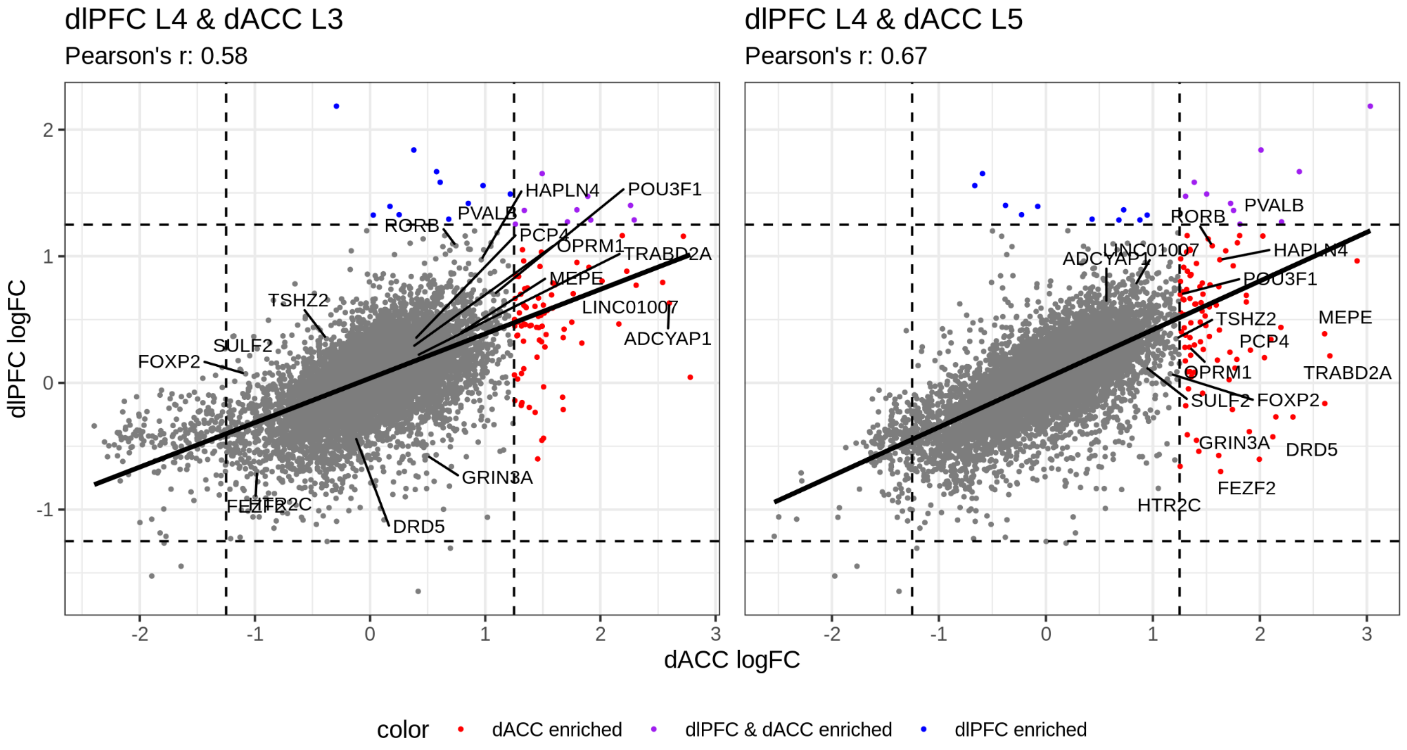

Supplementary Fig. 33. **Scatterplots comparing pseudobulked differential expression (DE) results per gene between dACC and dlPFC spatial domains.** Each scatterplot shows a comparison of the log fold-change (logFC) between one spatial domain in dACC and dlPFC (Huuki-Myers et al. 2024) SRT spatial domains. Each point is a gene. The *x*-axis of the first plot shows the logFC from enrichment model pseudobulked DE testing comparing Layer 3 to all other spatial domains for dACC, computing using `registration_wrapper()` (Pardo et al. 2022). The *y*-axis of the first plot shows the logFC from enrichment model pseudobulked DE testing comparing Layer 4 to all other spatial domains for dlPFC. The second plot compares L5 in the dACC to L4 in the dlPFC. The colors highlight genes that are classified as either dACC enriched (red), dlPFC enriched (blue), or both (purple), where the threshold is a logFC greater than 1.25. The number of genes in each category is as follows: L4 & L3) 77 dACC enriched & 11 dlPFC enriched & 9 dlPFC and dACC enriched and L4 & L5) 89 dACC enriched & 10 dlPFC enriched & 10 dlPFC and dACC enriched.

bioRxiv preprint doi: https://doi.org/10.1101/2025.07.14.664821; this version posted July 17, 2025. The copyright holder for this preprint (which was not certified by peer review) is the author/funder, who has granted bioRxiv a license to display the preprint in perpetuity. It is made available under aCC-BY 4.0 International license.

Supplementary Tables

Supplementary Table 1. Donor demographics. Demographics for the 10 neurotypical control donors including age, sex, diagnosis, postmortem interval (PMI), screening RNA integrity number (RIN) in prefrontal cortex (PFC), each assay performed, and the number of replicates included (Visium H&E, Visium-SPG, snRNA-seq).

Supplementary Table 2. nnSVG gene ranks. As described in **Methods**, table contains results from nnSVG analysis that were used to identify spatially variable genes (SVGs) in the SRT data. Columns include ENSEMBL ID, gene name, overall gene rank, mean gene rank across all capture areas, and the number of capture areas for which each gene was highly ranked (within top 1000 most variable genes).

Supplementary Table 3. Top 30 marker genes for snRNA-seq cell types. For each gene, a linear mixed-effects model was fit with counts pseudobulked across spots within a cell type to identify differences in expression enriched in one cell type compared to all other cell types using Student's *t*-test statistics.

Supplementary Table 4. Top 10 marker genes for snRNA-seq NMF patterns. For each NMF pattern, the top 10 genes contributing to the loadings matrix were selected as marker genes.

Supplementary Table 5. Top 50 marker genes for SRT spatial domains. For each gene, a linear mixed-effects model was fit with counts pseudobulked across spots within a spatial domain to identify differences in expression enriched in one domain compared to all other domains using Student's *t*-test statistics.

Supplementary Table 6. Top 50 pairwise markers comparing L6a and L6b SRT spatial domains. For each gene, a linear mixed-effects model was fit with counts pseudobulked across spots within a spatial domain to identify differences in expression enriched in spatial domain L6a compared to spatial domain L6b using Student's *t*-test statistics.
